# Genetic Diversity and Thermal Performance in Invasive and Native Populations of African Fig Flies

**DOI:** 10.1093/molbev/msaa050

**Published:** 2020-02-28

**Authors:** Aaron A Comeault, Jeremy Wang, Silas Tittes, Kristin Isbell, Spencer Ingley, Allen H Hurlbert, Daniel R Matute

**Affiliations:** m1 School of Natural Sciences, Bangor University, Bangor, Gwynedd, United Kingdom; m2 Department of Genetics, University of North Carolina at Chapel Hill, Chapel Hill, NC; m3 Department of Evolution and Ecology, University of California, Davis, Davis, CA; m4 Department of Biology, University of North Carolina at Chapel Hill, Chapel Hill, NC; m5 Faculty of Sciences, Brigham Young University, Hawaii, Laie, HI

**Keywords:** invasive species, genomics, genetic diversity, thermal performance, climate adaptation, *Zaprionus*

## Abstract

During biological invasions, invasive populations can suffer losses of genetic diversity that are predicted to negatively impact their fitness/performance. Despite examples of invasive populations harboring lower diversity than conspecific populations in their native range, few studies have linked this lower diversity to a decrease in fitness. Using genome sequences, we show that invasive populations of the African fig fly, *Zaprionus indianus*, have less genetic diversity than conspecific populations in their native range and that diversity is proportionally lower in regions of the genome experiencing low recombination rates. This result suggests that selection may have played a role in lowering diversity in the invasive populations. We next use interspecific comparisons to show that genetic diversity remains relatively high in invasive populations of *Z. indianus* when compared with other closely related species. By comparing genetic diversity in orthologous gene regions, we also show that the genome-wide landscape of genetic diversity differs between invasive and native populations of *Z. indianus* indicating that invasion not only affects amounts of genetic diversity but also how that diversity is distributed across the genome. Finally, we use parameter estimates from thermal performance curves for 13 species of *Zaprionus* to show that *Z. indianus* has the broadest thermal niche of measured species, and that performance does not differ between invasive and native populations. These results illustrate how aspects of genetic diversity in invasive species can be decoupled from measures of fitness, and that a broad thermal niche may have helped facilitate *Z. indianus*’s range expansion.

## Introduction

Populations of invasive species can experience extreme demographic histories. For example, processes such as bottlenecks, inbreeding, hybridization, and multiple introductions can all operate in populations of invasive species (Ellstrand and Schierenbeck 2000; Kolbe et al. 2004; Dlugosch and Parker 2008; Hovick and Whitney 2014; Barker et al. 2019). More generally, the impact of demography on levels of genetic variation in invasive populations has been a major focus in the field of invasion genetics since its inception >60 years ago (Baker and Stebbins 1965; Lee 2002; Dlugosch et al. 2015); especially with respect to its effect on invasive species’ ability to adapt to novel environments. Under the assumption that invasive species experience strong bottlenecks, and in some cases inbreeding, an erosion of genetic diversity is predicted to reduce fitness and impose constraints on a population’s ability to persist in and adapt to novel environments (Agashe and Bolnick 2010; Markert et al. 2010; Agashe et al. 2011). The fact that invasive species are frequently able to successfully colonize and adapt to novel environments, despite the negative consequences that a loss of diversity is expected to have on fitness (and by extrapolation, population growth rates), has led to the idea of the “genetic paradox of invasive species” (Allendorf and Lundquist 2003; Estoup et al. 2016).

Although studies have shown that some invasive populations experience a loss of genetic diversity relative to populations in the species’ native range (Tsutsui et al. 2000; Grapputo et al. 2005; Michaelides et al. 2018), these findings are frequently based on a small number of putatively neutral genetic markers. Others have shown that invasive populations can maintain high levels of genetic diversity through processes such as hybridization and multiple introductions stemming from different source populations in the species’ native range (Stepien et al. 2005; Lavergne and Molofsky 2007; Facon et al. 2008). The paradoxical nature of invasions has therefore been called into question (Dlugosch et al. 2015; Estoup et al. 2016).

In cases where invasive populations show less genetic diversity than native populations, at least two general arguments have been made against the idea of a genetic paradox in the invasive populations. First, lower genetic diversity at neutral loci is not equivalent to a loss of adaptive genetic variation: invasive populations may show reduced genetic variation at neutral loci but retain variation at loci that are important for maintaining fitness and adapting to novel environments (Dlugosch et al. 2015; Estoup et al. 2016). Second, invasive populations may not need to adapt to the habitats they are colonizing, therefore removing the paradox altogether (Estoup et al. 2016). For example, populations may adapt to human-altered or disturbed environments in their native range, thereby facilitating subsequent range expansions into “anthropogenic” environments (a process termed “anthropogenically induced adaptation to invade”; Hufbauer et al. 2012).

Theory predicts that changes in population size will affect both the overall amount and also the type of genetic variation found within a population. Genetic drift in small populations can, for example, lead to the fixation of weakly deleterious mutations segregating at low frequencies in source populations (Gillespie 1994; Marsden et al. 2016; Rogers and Slatkin 2017), and populations that are either increasing (e.g., a growing invasive population) or decreasing (e.g., bottlenecked populations at the front of a range expansion) in size are expected to fix novel beneficial or deleterious mutations, respectively, with higher probability than populations of constant size (Otto and Whitlock 1997). These theoretical expectations have important implications for the dynamics of adaptation (and maladaptation) during range expansions. We therefore require a better understanding of how biological invasions and range expansions affect genome-wide patterns of diversity and how these changes may alter the average fitness of individuals in invading populations.

When explicit links between genetic variation and adaptive phenotypic variation have not been made, a comparative approach can be used to gain insight into general effects that invasion has on genetic variation. First, genome-wide data can be used to gain a more nuanced understanding of patterns of genetic variation within populations found in invasive versus native parts of a species’ range. For example, genetic diversity varies across the genome (Langley et al. 2012; Dutoit et al. 2017) and genomic data from invasive and native populations could be used to test whether genetic diversity is consistently reduced across the genome. This approach has been used to suggest that the interaction between recombination rate, gene structure, and selection can generate conserved patterns of variation in genetic diversity across the genomes of different species (Langley et al. 2012; Dutoit et al. 2017). However, we do not know how invasion may alter genetic diversity at a genomic scale. Interspecific comparisons can therefore be used to generate a better understanding of if/how invasion affects the genome-wide distribution of genetic diversity.

Second, although studies of genetic variation in invasive species tend to focus on comparisons between invasive and native populations, comparisons between other invasive species and closely related noninvasive species can reveal whether the reduction in genetic diversity observed in an invasive population results in levels of genetic diversity that are below broadly observed levels. This type of comparison can be informative if populations that show comparable genetic diversity to the invasive population of interest also show evidence of contemporary adaptation to different environments. However, as mentioned above, summary statistics of genome-wide trends in genetic diversity may not reflect amounts of additive-genetic variation, and we require additional information to understand the effects of invasion on performance and adaptation in invasive populations.

From a phenotypic perspective, quantifying performance across different environments can be used to assess whether range expansions associated with biological invasions have affected the fitness of individuals in invasive populations. If inbreeding and small population size had led to a decrease in fitness, one prediction is that between-population crosses will display higher fitness than crosses carried out within an inbred population (i.e., heterosis; Oakley et al. 2019). More generally, if invasive populations suffer from inbreeding depression, we would predict that they will display lower fitness (or some measure correlated with fitness) relative to outbred populations that are found in the native portion of the species’ range (Oakley et al. 2019). Therefore, by combining genome-wide surveys of genetic variation with phenotypic measurements of performance, we can gain a better understanding of the processes affecting genetic variation during biological invasions and how those processes might affect the fitness of individuals in invading populations.

**Fig. 1.**
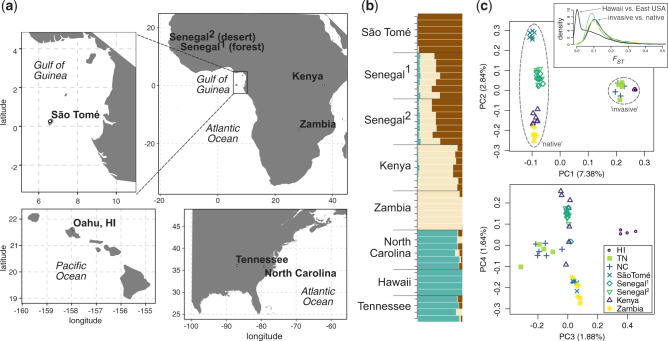
Collection locations across sub-Saharan Africa, eastern North America, and Hawaii (*a*) and genetic differentiation among *Zaprionus indianus* samples (*b* and *c*). (*a*) All collection locations are highlighted with bold type. *Zaprionus indianus* was sampled from all locations, *Z. tuberculatus* was sampled from São Tomé and Senegal (forest site), *Z. africanus* from São Tomé and Kenya, and *Z. inermis*, *Z. tsacasi*, *Z. taronus*, and *Z. nigranus* from São Tomé. Invasive populations of *Z. indianus* are differentiated from populations in their native range (*b* and *c*), with the strongest genetic differentiation between invasive and native populations (*c*; PC1) and among African populations (*c*; PC2). Invasive populations in the eastern United States and Hawaii also show weak differentiation (*c*; PC3). The inset in the top right of (*c*) shows the genome-wide distribution of differentiation (*F*_ST_; 5-kb genomic windows) between invasive *Z. indianus* and each of the four African populations (Senegal sample locations grouped as one population), and also between eastern United States and Hawaiian samples (black line).

Here, we analyze whole-genome sequences collected from 93 individuals sampled across 7 species and 16 populations of African fig fly (genus *Zaprionus*; fig. 1) to test whether invasive populations of *Z. indianus* are outliers with respect to the genetic variation they harbor. We also use these species to quantify the genome-wide diversity landscape and test whether it has been altered in invasive populations of *Z. indianus*. Finally, we estimate thermal performance curves for 21 populations across 13 species of African fig fly to test whether there is any general relationship between levels of genetic variation and performance across a broad range of temperatures. We find that invasive populations of *Z. indianus* have lower genetic diversity than populations in their native range, and that both the presence or absence of genes and local recombination rate affect amounts of genetic diversity. However, genetic diversity in invasive populations tends to remain as high or as higher than in noninvasive species of *Zaprionus*. Estimates of thermal performance curves show that *Z. indianus* has the broadest thermal niche of the species we tested, and despite lower genetic diversity, invasive populations of *Z. indianus* do not show reduced performance relative to populations in their native range. Together, our results suggest that a broad thermal niche may have facilitated the range expansion of *Z. indianus* and that large populations (and high genetic diversity) in *Z. indianus*’s native range may have acted to buffer invasive populations against critical losses of genetic diversity.

## Results

We estimated genetic differentiation among populations of *Z. indianus* to test whether genetic affinities between invasive and native populations suggested potential sources of the invasion (fig. 1 and Supplementary Material online). Population assignment (fig. 1*b*), principal component analysis (fig. 1*c*), and genome-wide estimates of differentiation (*F*_ST_) showed that invasive populations were closely related to one another and genetically differentiated from African populations. Despite broad geographic sampling in Africa, these analyses did not indicate that invasive populations were more closely related to any one African population (median *F*_ST_ ranged from 0.14 to 0.19 between invasive and African populations), as would be expected if they were recently derived from that population, or an unsampled, but closely related one. Below, we focus on aspects of genetic diversity within each population rather than differentiation among them.

### Invasive Populations of *Z. indianus* Have Less Genetic Diversity than Native Populations

A central tenet of the genetic paradox of invasive species is that relatively few individuals colonize invasive parts of their range and that these populations are subject to a loss of genetic diversity, and potentially to inbreeding (Allendorf and Lundquist 2003; Estoup et al. 2016). We tested for relatedness and inbreeding in our samples, but found no evidence that any of the individuals we sampled were closely related (all kinship coefficients estimated within populations <0.017). There was also no evidence for inbreeding in the invasive range of *Z. indianus* relative to its native range (*F*_1,17_ = 2.37; *P *=* *0.14; supplementary fig. S1, Supplementary Material online).

**Fig. 2.**
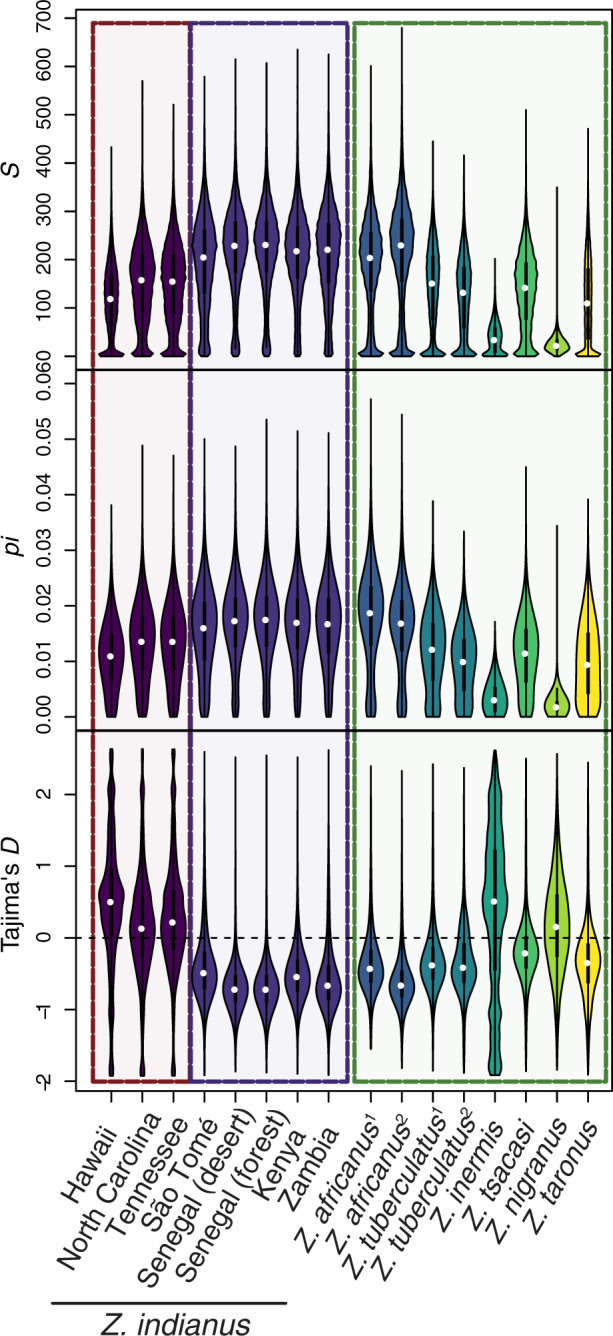
Estimates of genetic diversity summarized across 5-kb genomic windows for each population included in this study. Colored backgrounds group populations as invasive *Zaprionus indianus* (three leftmost violins), native *Z. indianus* (five central violins), and other species (eight rightmost violins). See supplementary figure S2, Supplementary Material online, for estimates for windows overlapping with BUSCO annotations and supplementary table S4, Supplementary Material online, for estimates in all subsamples from populations where we sampled more than four individuals. Superscript 1 indicates population of *Z. africanus* or *Z. tuberculatus* sampled from São Tomé and superscript 2 indicates population of *Z. africanus* from Kenya or *Z. tuberculatus* from Senegal (forest site).

We computed nucleotide diversity (*π*), the number of segregating sites (*S*), and Tajima’s *D* in nonoverlapping 5,000-bp windows across the genome for 16 populations across 7 species of *Zaprionus*. Among populations of the invasive *Z. indianus*, populations sampled in the invasive part of the species’ range display significantly less genetic diversity than populations sampled in their native range: the median number of segregating sites (*S*) in 5-kb genomic windows was between 120 and 160 across North American and Hawaiian populations (5% empirical quantiles: 0–10 SNPs; fig. 2 and supplementary table S4, Supplementary Material online) and 206–233 across African populations (5% empirical quantiles: 21–37 SNPs; fig. 2 and supplementary table S4, Supplementary Material online). We observed similarly low genetic diversity in invasive, relative to native, populations of *Z. indianus* when we restricted our analysis to genomic windows that overlap an annotated BUSCO gene (supplementary fig. S2 and table S5, Supplementary Material online). These results are in line with studies in other systems that have shown lower genetic diversity in invasive populations than in native populations of invasive species (Grapputo et al. 2005; Michaelides et al. 2018).

Tajima’s *D* also varied across populations of *Z. indianus*, with invasive populations showing positive median genome-wide estimates of Tajima’s *D* (0.13–0.5), consistent with a recent contraction in population size, and native populations showing negative median estimates of Tajima’s *D* (−0.48 to −0.72), consistent with range expansion (fig. 2 and supplementary table S4, Supplementary Material online). A positive genome-wide estimate of Tajima’s *D* in the invasive populations of *Z. indianus*, together with lower genetic diversity relative to the native populations, points to an active loss of lost genetic variation occurring in the invasive populations during their range expansion. However, because the ancestral population that gave rise to the invasive populations is unknown, additional population samples and more thorough demographic analyses are required to test the specific demographic scenarios driving the patterns we observe with respect to genetic diversity in these populations.

### Genetic Diversity in Invasive Populations of *Z. indianus* Is Not Exceptionally Low

Although levels of genetic diversity were reduced in invasive populations of *Z. indianus*, we found that these populations still harbor as much, or more, genetic diversity than naturally occurring noninvasive species of *Zaprionus* (fig. 2). For example, *S* within populations of *Z. tuberculatus* and *Z. tsacasi* (median *S *=* *141 and 143, respectively) is comparable to *S* in invasive populations of *Z. indianus* (median *S *=* *120–140). By contrast, populations of *Z. nigranus*, *Z. taronus*, and *Z. inermis* all show markedly less genetic diversity (median *S *=* *23, 106, and 35, respectively) than any of the *Z. indianus* populations we sampled. The broadly distributed species *Z. africanus* (the closest relative to *Z. indianus* included in this study) showed amounts of genetic diversity comparable with those in native populations of *Z. indianus*. It is also worth noting that the level of genetic diversity we observe in invasive and native populations of *Z. indianus* is similar to the distantly related invasive Drosophilid fly *Drosophila suzukii* (nucleotide diversity = 0.88–2.28%; Adrion et al. 2014), but somewhat greater than estimates for *D. melanogaster* (nucleotide diversity across multiple studies and populations ranges from 0.1% to 0.9%; Begun and Aquadro 1992; Langley et al. 2012; Pool et al. 2012).

Similar to comparisons among populations of *Z. indianus*, we recovered the same relationship when restricting our analysis to genomic windows that overlap an annotated BUSCO gene (supplementary tables S4 and S5, Supplementary Material online). Patterns of genetic diversity across populations of *Z. indianus* and other species of *Zaprionus* suggest that, under the assumption that the amount of genetic diversity in noninvasive species is a reasonable proxy for the amount of genetic diversity a population can have without negative fitness effects, high levels of genetic diversity in the native range of invasive species may buffer invasive populations against losses of diversity below levels that would negatively affect fitness or adaptive potential.

### Genetic Diversity Is Affected by Genome Architecture

Although population bottlenecks occurring in invasive populations are expected to lower genetic diversity broadly across the genome (Hyten et al. 2006; Ellegren and Galtier 2016), other processes can act locally within the genome and either reduce (e.g., selective sweeps; Charlesworth et al. 1997) or maintain genetic diversity (e.g., balancing selection; Aguilar et al. 2004; Charlesworth 2006; Lindtke et al. 2017). The fitness consequences of diversity across loci are also not expected to be equal. Genetic diversity therefore varies greatly across the genome and the interaction between selection and recombination rate can lead to a genome-wide “landscape” of genetic diversity that is correlated with aspects of genome architecture such as recombination rate or gene density (Begun and Aquadro 1992; Nachman 2001; Burri et al. 2015; Dutoit et al. 2017). We generated gene annotations and used estimates of recombination rates to test the relationship between genetic diversity in invasive and native populations of *Z. indianus* and these features of the genome.

Across all genomic windows, we found that the amount of genetic diversity (*S*) within a given genomic window varied depending on whether that window overlapped-with, was adjacent-to, or was distant-from an annotated gene (population level comparisons: generalized linear models [GLMs]: all *P *<* *0.0001). Genetic diversity tended to be lower in windows that overlap an annotated gene compared with windows that were either within or outside of 5-kb from an annotated gene (12 of 15 populations; binomial test: *P *=* *0.035; supplementary fig. S3, Supplementary Material online). Three populations did not follow this trend: the two *Z. africanus* populations and the *Z. tuberculatus* population sampled from Senegal. For populations that had less diversity in windows that overlapped an annotated gene, mean diversity tended to be 3.5–21.5% lower than in windows within 5-kb of an annotated gene, and 0.6–32.9% lower than in windows further than 5-kb from an annotated gene (supplementary table S6, Supplementary Material online). We did not, however, observe an interaction between invasion status (invasive *Z. indianus*, native *Z. indianus*, or noninvasive *Zaprionus*) and window-location relative to annotated genes and the median amount of genetic diversity (*S*) observed across windows (GLM: *P *=* *0.99). The amount of genetic diversity within a genomic region is therefore affected by the presence (or absence) of genes, but broad scale differences in diversity between “genic” and “nongenic” regions is not systematically altered—for example, due to selection preferentially maintaining genetic diversity in or around genes—during the course of invasion.

**Fig. 3.**
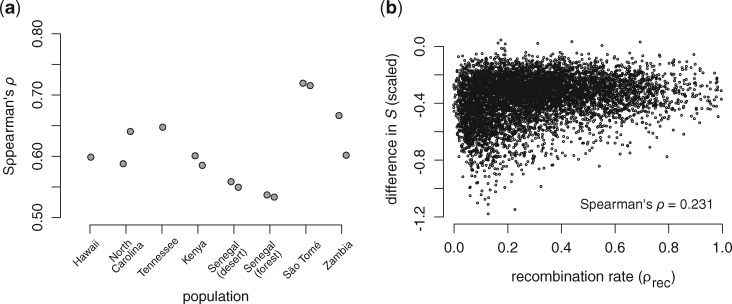
Correlations between genetic diversity (the number of segregating sites: *S*) and recombination rate across populations of *Zaprionus indianus* (*a*). Correlations did not systematically differ between populations in the invasive and native regions of the species’ range. However, the mean difference in diversity for a given genomic window was positively correlated with recombination rate (*b*). Populations with two points in (*a*) represent populations where we sampled more than four individuals and estimated *S* using two independent random subsamples of those individuals. In (*b*), the difference in *S* (mean in invasive populations−mean in native populations) was scaled by mean levels of diversity for a given recombination rate quantile.

We next explored the relationship between genetic diversity (*S*) and mean estimates of population-scaled recombination rates in 5-kb genomic windows (Materials and Methods). Consistent with previous studies (Begun and Aquadro 1992; Begun et al. 2007; Burri et al. 2015; Dutoit et al. 2017; Samuk et al. 2017), *S* was positively correlated with recombination rate across genomic windows in all *Z. indianus* populations (all Spearman’s *ρ *> 0.5; fig. 3*a*). The strength of this correlation did not systematically differ between invasive and native populations of *Z. indianus* (linear model: *F*_1,12_ = 0.1025; *P *=* *0.7543); however, the mean difference in *S* between invasive and native populations of *Z. indianus* was correlated with local recombination rate (analysis of windows with mean *S* between 150 and 300 in *Z. indianus*’s native range; fig. 3*b*). This correlation was weak when analyzing the raw difference in *S* (Spearman’s *ρ*  =  0.071; supplementary fig. S4, Supplementary Material online), but was modest when the difference in *S* was scaled by mean *S* for genomic windows binned by recombination rate quantiles (Spearman’s *ρ*  =  0.231; fig. 3*b*). This finding shows that during invasions, the loss of diversity is not uniform across the genome, but is greater in genomic regions experiencing lower recombination rates.

### Invasion Alters the Genome-Wide “Landscape” of Genetic Diversity

**Fig. 4.**
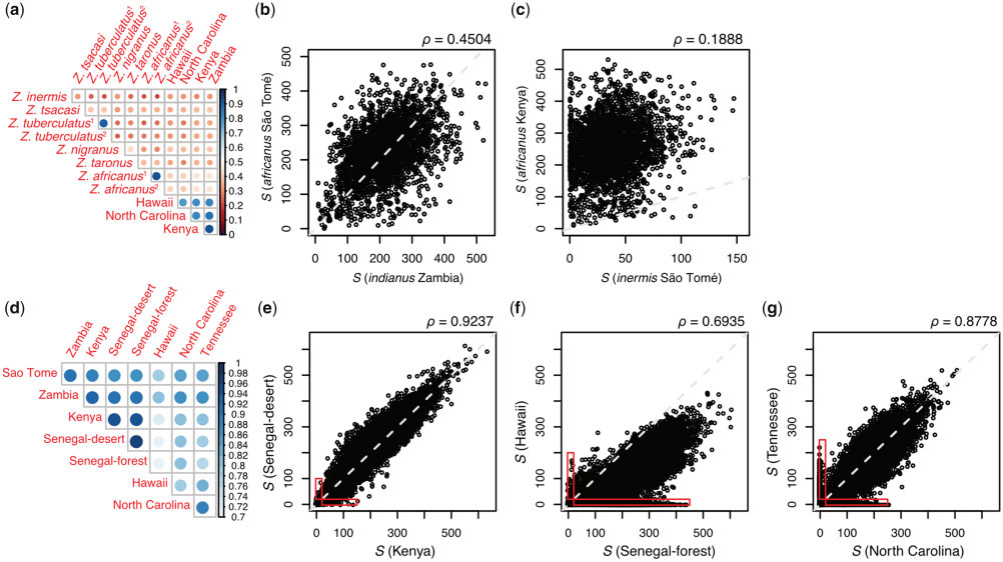
Genetic diversity (*S*) is correlated across regions of the genome containing annotated single-copy orthologs in interspecific comparisons (*a*). The closely related species *Zaprionus africanus* from São Tomé and *Z. indianus* from Zambia had the strongest interspecific correlation in *S* (*b*; see fig. 4*a* for phylogeny) and the distantly related species *Z. africanus* from Kenya and *Z. inermis* from São Tomé had the weakest correlation in *S* (*c*). Genetic diversity is strongly correlated in all pairwise comparisons between populations of *Z. indianus* (*d*). (*e* and *f*) Data from the strongest and weakest between-population correlations for *Z. indianus*, and (*g*) shows the strongest correlation between invasive populations of *Z. indianus*. Red rectangles in (*e*) through (*g*) highlight genomic windows that have low diversity in one population (fewer than ten segregating SNPs), but higher diversity in the other (more than ten segregating SNPs).

**Fig. 5.**
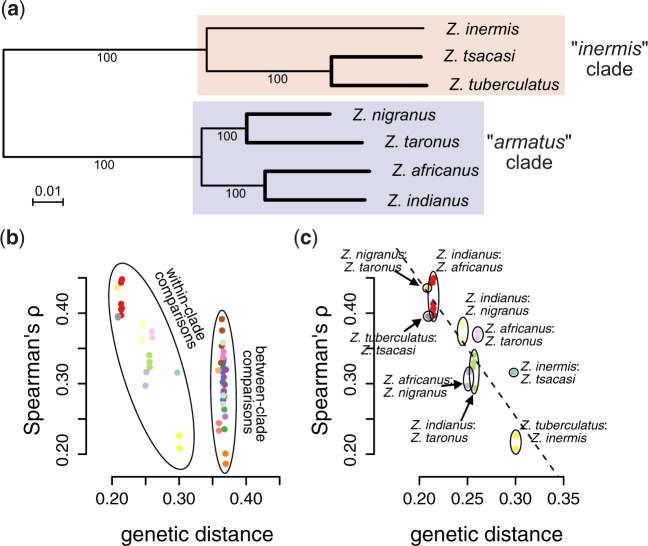
The correlation of genetic diversity (*S*) across the genomes of different species (*a*) decreases with increasing genetic distance (*b* and *c*). Maximum likelihood phylogeny in (*a*) was estimated with RAxML run on an alignment of 1,709 BUSCO genes annotated across all seven species’ genomes. Correlation coefficients decreased with increasing genetic distance (Mantel *r* = −0.816; *b*), with this pattern being particularly evident for within-clade comparisons (highlighted in *c*).

As a second test of whether the genome-wide distribution of diversity is altered during biological invasions, we compared genetic diversity between species in genomic windows that spanned annotated BUSCO genes. Consistent with a genome-wide landscape of genetic diversity, we found that genetic diversity (*S*) within annotated BUSCO genes was correlated between populations and species of *Zaprionus* (fig. 4; see supplementary fig. S5 for randomization test, Supplementary Material online). As expected, correlations in *S* were weaker for interspecific comparisons than for intraspecific comparisons (fig. 4*a*). The strongest interspecific correlation we observed was between *Z. indianus* from Zambia and *Z. africanus* from São Tomé (Spearman’s *ρ*  =  0.4504; fig. 4*b*) and the weakest was between *Z. inermis* from São Tomé and *Z. africanus* from Kenya (Spearman’s *ρ*  =  0.1888; fig. 4*c*). Across all pairwise comparisons, there was a significant negative relationship between the correlation in *S* across the genome and the genetic distance between the species being tested (Mantel *r* = −0.816; *P *=* *0.0001; fig. 5*a* and *b*; pairwise sequence differences ranged from 4.3% to 13.7% for the species compared here; see supplementary table S7, Supplementary Material online). This trend was even stronger among comparisons between more closely related species pairs (i.e., “within-clade” comparisons; Mantel *r* = −0.83; fig. 5*c*). The pattern of weaker correlations in *S* between more genetically diverged species is consistent with evolution of aspects of genome architecture (e.g., intron size, recommendation rates, and/or transposable element evolution) altering the genome-wide landscape of diversity; however, explicit tests of these mechanisms are still needed.

As with interspecific comparisons restricted to BUSCO windows, genetic diversity (*S*) was significantly correlated across all genomic windows between populations of *Z. indianus* (fig. 4*d*). Among African populations of *Z. indianus*, this was not a function of how closely related two populations were: geographically distant populations in Senegal and Kenya or Senegal and Zambia (fig. 1) show among the strongest correlations in *S* (Spearman’s *ρ*  >  0.885; fig. 4*d* and *e*). In general, the correlation in *S* was lower when a native and an invasive populations were being compared versus comparisons made among native populations (maximum *ρ*  =  0.865, minimum *ρ  *=  0.694; fig. 4*d* and *f*). Among invasive populations, North Carolina and Tennessee populations showed the strongest correlation in *S* (Spearman’s *ρ*  >  0.878; fig. 4*g*); however, this correlation was lower than the five strongest correlations, all observed between African populations.

In contrast to *S*, Tajima’s *D* was either weakly correlated or uncorrelated between species (supplementary fig. S5*a*, Supplementary Material online), and there was no relationship between the strength of correlation in Tajima’s *D* and phylogenetic distance (*P *>* *0.1). By contrast, Tajima’s *D* was significantly correlated across genomic windows between all populations of *Z. indianus* (*P *<* *0.001; supplementary fig. S5*b*, Supplementary Material online). However, Tajima’s *D* was more weakly correlated than *S* (compare supplementary fig. S5*b*, Supplementary Material online with fig. 4*d*). As with *S*, the strongest correlations in Tajima’s *D* were between populations sampled in Africa, consistent with a shared demographic history in the native part of *Z. indianus*’ range (supplementary fig. S5*b* and *c*, Supplementary Material online). By contrast, Tajima’s *D* was only weakly correlated in between-continent comparisons (strongest between-continent *ρ*  =  0.192; weakest *ρ*  =  0.0167; supplementary fig. S5*d*, Supplementary Material online) and moderately correlated between the two populations sampled in the eastern United States (Tennessee and North Carolina; *ρ*  =  0.2002; supplementary fig. S5*e*, Supplementary Material online). The fact that Tennessee and North Carolina have both been recently colonized during *Z. indianus*’s expansion into North America (∼20 years ago; Gibert et al. 2016), yet show relatively modest correlations in Tajima’s *D*, illustrates how demographic events can rapidly alter the frequency of alleles across the genome and suggests that these two locations are experiencing semi-independent colonizations, rather than an expansion driven by a single panmictic population.

**Fig. 6.**
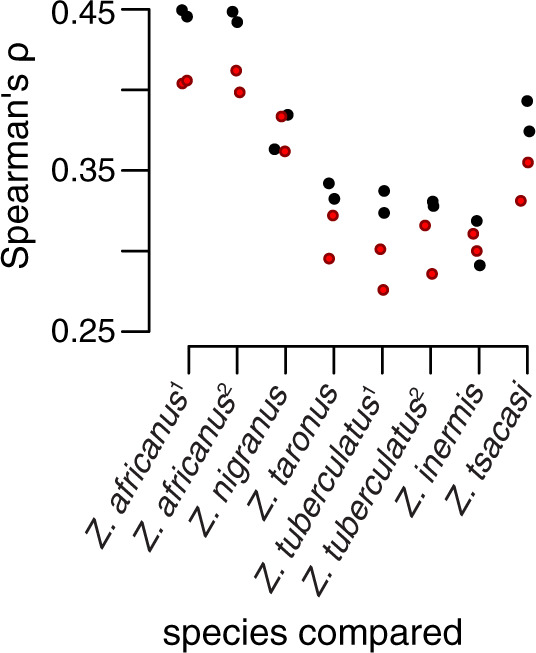
The correlation in genetic diversity (*S*) between invasive populations of *Zaprionus indianus* and other species of *Zaprionus* (dark red points) is weaker than the correlation in *S* between native populations of *Z. indianus* and other species of *Zaprionus* (black points).

The correlation of genetic diversity (*S*) between *Z. indianus* and other species of *Zaprionus* (figs. 4 and 5) allowed us to test whether this correlation is maintained following biological invasion. We find that interspecific correlations in *S* are weaker between invasive populations and other species than between native populations and other species (six of eight species:population comparisons; Fisher’s exact test: *P *=* *0.0093; fig. 6). When we restrict this same analysis to include one population per species, the correlation in genetic diversity remains weaker between invasive populations of *Z. indianus* and other *Zaprionus* species than between native populations of *Z. indianus* and other *Zaprionus* in four of the six species-level comparisons (Fisher’s exact test: *P *=* *0.03). Native populations of *Z. indianus* also never showed consistently weaker correlations than invasive populations (fig. 6). These findings illustrate how demographic and selective processes associated with biological invasions can both reduce genetic diversity and alter the genome-wide distribution of that diversity.

### Genetic Diversity Is Correlated with Thermal Niche Breadth

Genetic diversity can, in some cases, be positively correlated with measures of performance or fitness (Reed and Frankham 2003; Briskie and Mackintosh 2004; Leimu et al. 2006; Markert et al. 2010). We tested whether genetic diversity and fitness/performance were correlated using estimates of genetic diversity summarized above and thermal performance curves.

We estimated thermal performance curves using a hierarchical Bayesian framework (Tittes et al. 2019; Supplementary Material online). Parameters summarizing performance were estimated at the population level, allowing us to make comparisons between populations of *Z. indianus* sampled from three populations from their invasive range in eastern North America (Florida, North Carolina, and New York) and their native range in Africa (São Tomé, Senegal, Kenya, and Zambia).

**Fig. 7.**
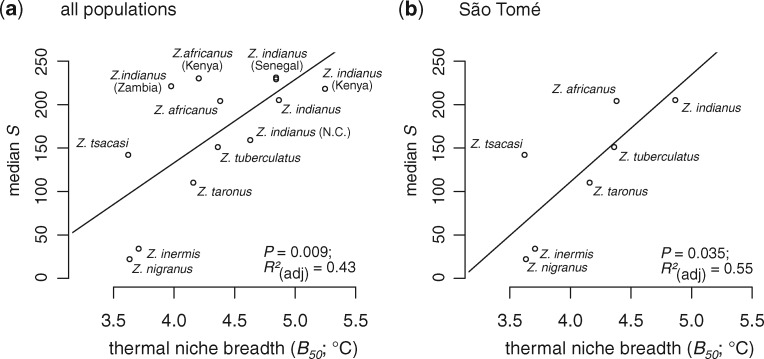
Populations and species with a larger thermal niche breadth (*B*_50_) also harbor more genetic diversity (median number of segregating sites; *S*). Results for all populations (*a*) and for only the seven species sampled on the island of São Tomé (*b*) are shown. Species names that are not labeled with a location in (*a*) are from São Tomé. Results from linear models testing the relationship between thermal niche breadth and genetic diversity are reported in the bottom right of each panel.

Among populations of *Zaprionus* species with estimates of genetic diversity and thermal performance, thermal niche breadth (*B*_50_) and the total area of the thermal performance curve (*A*_c_) were positively associated with genetic diversity (fig. 7*a*; linear models: *B*_50_: *F*_1,11_ = 10.1; *P *=* *0.009; *A*_c_: *F*_1,11_ = 7.402; *P *=* *0.020; note that *A*_c_ and maximum fitness parameters were strongly correlated; *r *=* *0.99). By contrast, there was no relationship between levels of genetic diversity and thermal optima (*F*_1,11_ = 1.625, *P *=* *0.23). Linear models fit to our complete data set should however be interpreted with caution because of phylogenetic nonindependence and uneven sampling across species (e.g., we sampled multiple populations of *Z. indianus*, *Z. africanus*, and *Z. tuberculatus*). We therefore also fit models that only included a single population from each of the seven species sampled from São Tomé (fig. 7*b*). This reduced data set still found support for a positive relationship between genetic diversity and thermal niche breadth (*B*_50_: *F*_1,5_ = 8.244; *P *=* *0.035; *R*^2^ = 0.55), but no relationship between genetic diversity and *A*_c_ or thermal optimum (both *P *>* *0.1). These results are consistent with genetic diversity being higher in species that are able to exploit a broad range of thermal environments, potentially due to these species maintaining large population sizes.

### Performance Is Not Reduced in Invasive Populations

**Fig. 8.**
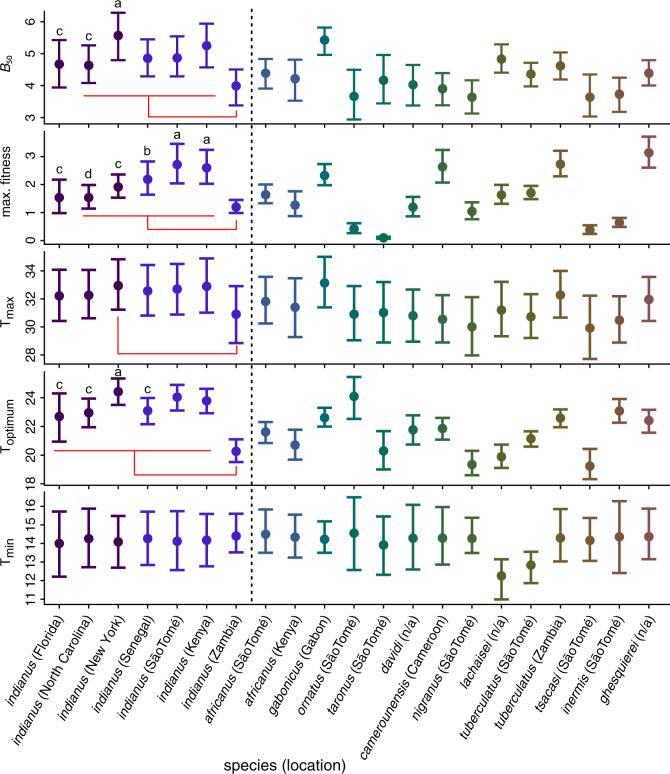
Median and 95% credible intervals for parameters estimated by jointly fitting performance curves to thermal performance data collected at mean temperatures of 13.9–28.9 °C. Populations of *Zaprionus indianus* are shown to the left of the dashed line and all other species of *Zaprionus* to the right. For populations of *Z. indianus*, parameter estimates that had a probability >0.95 of being different from one another in pairwise comparisons (analogous to *P *<* *0.05) are denoted by letters (“a” > “c” and “d,” and “b” > “d”). Red bars indicate significant differences between the population of *Z. indianus* from Zambia and other populations of *Z. indianus*.

Among the seven species from which we collected population genomic data, *Z. indianus* tended to have the highest estimated *B*_50_ (fig. 7). We tested the generality of this pattern by estimating thermal performance curves for an additional 6 species and 13 populations of *Zaprionus*. In this data set, populations of *Z. indianus* from both their native and their invasive ranges consistently have larger estimates of *B*_50_ than other populations and species (fig. 8). Two exceptions to this trend are that the *B*_50_ of *Z. indianus* from Zambia is more similar to *Z. africanus* than other populations of *Z. indianus*, and the *B*_50_ of *Z. gabonicus* is similar to populations of *Z. indianus* (fig. 8).

We next tested whether invasive and native populations of *Z. indianus* differed in maximum fitness, as measured by the total number of adult progeny produced by a single pair of flies, at their optimal temperature (“stretch” parameter reported in Tittes et al. 2019; “max. fitness” in fig. 8). African populations of *Z. indianus* from Kenya and São Tomé have higher maximum fitness than invasive populations from Florida, New York, and North Carolina, whereas the population from Senegal only had higher maximum fitness than the invasive population sampled from North Carolina (fig. 8). All populations of *Z. indianus* other than those from Florida and North Carolina had higher maximum fitness than the population from Zambia (fig. 8). The lower maximum fitness observed in invasive populations, compared with populations from Kenya and São Tomé, could be explained by a loss of genetic diversity having negative effects on fertility, fecundity, or survivorship. However, if a loss of genetic diversity underlies a decrease in maximum fitness, one prediction is that these effects would be particularly pronounced at stressful temperatures (i.e., near the minimum and maximum acceptable temperatures), thereby reducing the breadth of the performance curve. This was not the case, as estimates of thermal minima and maxima did not consistently differ between invasive and native populations (*T*_min_ and *T*_max_ parameters, respectively; fig. 8). There was also no evidence that maximum fitness in invasive populations (fig. 8) was different from that in the African populations collected in Senegal, despite the latter having high levels of genetic diversity. Therefore, we did not find consistent evidence that the loss of diversity associated with *Z. indianus*’s range expansion into North America has reduced the generally high performance that this species displays across a broad range of temperatures.

In addition to comparisons among populations of *Z. indianus*, populations of *Z. indianus* from Kenya and São Tomé both had maximum fitness equal to or greater than the 12 other species of *Zaprionus* we measured (fig. 8). The population from Senegal had a similarly high estimate of maximal fitness compared with all other species, except *Z. ghesquierei*, which showed a higher maximal fitness than all three invasive North American populations of *Z. indianus*, as well as *Z. indianus* from Senegal and Zambia (fig. 8). Three other species were estimated to have a maximal fitness that was greater than *Z. indianus* from at least two of the three North American populations: *Z. gabonicus*, *Z. camerounensis*, and *Z. tuberculatus*. This result suggests that the range of temperatures over which a species or population can maintain relatively high fitness (e.g., *B*_50_) is a more important factor differentiating *Z. indianus* from noninvasive species of *Zaprionus* than the absolute maximal fitness/performance that individuals in a population can achieve.

## Discussion

Identifying the genetic changes that are associated with biological invasions, along with effects those changes have on performance and fitness, is important for understanding the evolutionary controls acting on invasive species (Lee 2002; Lawson Handley et al. 2011; Stapley et al. 2015; Estoup et al. 2016). We have shown that the range expansion of the invasive African fig fly, *Z. indianus*, has resulted in a loss of diversity in invasive populations, but that levels of diversity in invasive populations remain as high or higher than those observed in other species in the genus *Zaprionus*. The level of genetic diversity we observe in *Z. indianus* (fig. 1 and supplementary table S4, Supplementary Material online) is also similar to those reported for the invasive species *D. suzukii* (Adrion et al. 2014) and somewhat higher than values that have been reported in populations of *D. melanogaster* (Begun and Aquadro 1992; Langley et al. 2012; Pool et al. 2012). In the case of *D. melanogaster*, populations in the invasive (or nonnative) part of the species’ range show geographic and temporal changes in allele frequency that are consistent with adaptation to novel environments (Bergland et al. 2014; Adrion et al. 2015). These interspecific comparisons illustrate how invasive populations with lower genetic diversity than native populations can still have sufficient amounts of genetic diversity to facilitate adaptation to novel environments. Attributes of successful invasive species’ biology, such as a generalist life history (Sakai et al. 2001; Allendorf and Lundquist 2003), can therefore help them maintain genetic diversity and avoid the “genetic paradox” that may occur under more extreme or stereotyped range expansions (i.e., severe bottleneck with relatively few colonizing individuals).

A number of evolutionary mechanisms that act to increase (or maintain) genetic diversity in invasive populations could also help explain the high levels of genetic diversity in invasive populations of *Z. indianus*. For example, multiple introductions from distinct populations in the native range and admixture can both act to generate high levels of genetic diversity in invasive populations (Stepien et al. 2005; Dlugosch and Hays 2008; Rosenthal et al. 2008; Barker et al. 2017). High levels of genetic diversity in native populations of *Z. indianus* (fig. 2) may also act to buffer invasive populations against a loss of diversity, but without knowing the source population(s) of invasive *Z. indianus* we are not able to test this hypothesis.

In addition to comparing overall levels of genetic diversity, we were able to use interspecific comparisons to test if and how invasion alters how that genetic diversity is distributed across the genome. Correlated levels of genetic diversity have been shown between a pair of divergent bird species (Dutoit et al. 2017), but to the best of our knowledge, this has not been tested in a phylogenetic context until now. We identified and used the correlation in diversity between species (fig. 5*b* and *c*) to show that the genome-wide landscape of genetic diversity is altered in invasive, relative to native, populations of *Z. indianus* (fig. 6). We also show that the difference in the amount of genetic diversity between invasive and native populations of *Z. indianus* tends to be larger in regions of the genome with low recombination rates (fig. 3*b*). Previous studies have reported a similar pattern with respect to lower genetic diversity on the *X*-chromosome in non-African populations of *D. simulans* (Begun and Whitley 2000; Schöfl and Schlötterer 2006). We have yet to identify the scaffolds that compose the sex chromosomes in the genome assemblies we report here, but larger reductions in diversity on the *X*-chromosome relative to the autosomes is likely to contribute to the pattern of reduced genetic diversity in regions of low recombination. One mechanism that could explain this pattern is linked selection (Nordborg et al. 1996; Charlesworth et al. 1997; Begun and Whitley 2000), as selection will have a larger spatial effect on levels of genetic diversity in regions of the genome with low recombination rates. Future work is needed to identify both the chromosome structure of *Zaprionus* species and the regions of the genome potentially subject to selection in invasive populations of *Z. indianus*.

Despite the differences in genetic diversity between invasive and native populations of *Z. indianus*, we found that individuals from both regions do not differ in their ability to maintain high performance across a broad range of thermal environments (fig. 8). This finding is in contrast with studies that have found a positive correlation between genetic diversity and measures of fitness (Reed and Frankham 2003; Leimu et al. 2006; Markert et al. 2010; but see Lammi et al. 1999).

The broad thermal niche of *Z. indianus* populations across their native and invasive ranges may help explain this species’ high level of genetic diversity and success as an invasive species. For example, if *Z. indianus* evolved a broad thermal niche prior to expanding their range out of Africa, this could have led to large and broadly distributed populations that would build up high levels of genetic diversity. If alleles underlying thermal performance traits became fixed (or present at a high frequency) in native populations prior to range expansion, reductions in genetic diversity would not remove those high-frequency adaptive alleles. This scenario is related to the idea that adaptation to anthropogenically disturbed environments in the native portion of a species’ range can facilitate subsequent range expansions (Lee and Gelembiuk 2008; Hufbauer et al. 2012). Although the timing of adaptive trait evolution in populations of *Z. indianus*, relative to their range expansion, is not known, anecdotal evidence from collecting *Zaprionus* across locations in their native Africa suggest that *Z. indianus* possess a generalist and “invasive-like” life history (personal observation). For example, we collected *Z. indianus* near cities or rural human settlements in rainforest, savannah, and desert environments, and *Z. indianus* is among the first and most abundant species to visit fruit traps across these environments. If the traits that underlie a broad thermal niche evolved in African populations prior to range expansion, then lower diversity in invasive populations may not have negatively affected the fitness of *Z. indianus* in the invasive part of their range.

Estimates of thermal performance curves suggest that, despite the climate being markedly cooler at sample sites in northeastern North America (e.g., North Carolina and New York) than at African sites, populations of *Z. indianus* in North America have not evolved to tolerate more temperate climates that African populations (estimated minimum temperature where fitness falls to 0 does not differ between any population of *Z. indianus*; *T*_min_ parameter in fig. 8). Thermal niche breadth also does not systematically differ between *Z. indianus* from New York, São Tomé, and Kenya, and these populations have significantly wider thermal niches than almost all other *Zaprionus* species or population in our data set (all *P *<* *0.05 in pairwise comparisons; fig. 8). Together, these results indicate that the thermal niche of North American and African *Z. indianus* has not evolved to be different, and that the loss of genetic diversity in invasive populations has not reduced the broad range of temperatures across which *Z. indianus* is capable of maintaining relatively high fitness (i.e., *B*_50_; fig. 8).

An alternate interpretation for the lack of differences in thermal performance curves between invasive and native populations of *Z. indianus* is that the lower level of genetic diversity in North American populations of *Z. indianus* is constraining potentially adaptive niche evolution in these populations. Comparing levels of diversity that segregate in *Z. indianus* to those in other Drosophilid populations suggest this is unlikely: invasive populations of *Z. indianus* still harbor as much or more genetic diversity than other species of *Zaprionus* and *Drosophila*. However, future work testing levels of additive-genetic variation for traits involved in thermal tolerance are needed to quantify the adaptive potential of the different populations and species.

Traits associated with thermal performance are only one suite of traits potentially under selection during colonization and range expansion into novel environments. Other traits such as competitive ability (Blossey and Notzold 1995) and the ability to utilize a broad range of other habitats (Lee and Gelembiuk 2008) can affect the success of invasive species. Of particular note is the fact that we quantified thermal performance on a single food resource (standard cornmeal media for *Drosophila*). Measuring performance in the same populations and species analyzed here, across a wide range of diets, would be useful to determine the extent to which invasive populations of *Z. indianus* have adapted to successfully colonize the broad range of environments they now occupy.

In conclusion, we have shown that genetic diversity in invasive populations of *Z. indianus* is lower than in native populations (fig. 2), and that the genome-wide distribution of genetic variation is perturbed in invasive populations (figs. 3*b* and 4–6). These results provide a more nuanced understanding of how range expansions associated with invasion can alter levels of genetic variation across the genome. Despite these effects on genetic diversity, invasive populations of *Z. indianus* maintain as much or more genetic diversity than noninvasive congeneric species (fig. 2), and both invasive and native populations of *Z. indianus* are capable of maintaining high fitness across a broad range of thermal environments (figs. 7 and 8). These results show how measures of fitness in invasive species can be decoupled from genetic diversity. They also suggest that adaptation to an “invasive” niche prior to range expansion may be a more important event in the evolutionary history of an invasive lineage than the demographic events that take place during subsequent expansion of their range into novel environments. Future work identifying the evolutionary history of invasive traits (and their underlying genetic variation) will be central to our understanding of the factors controlling the success and spread of invasive species.

## Materials and Methods

Detailed methods can be found in the Supplementary Material online.

### Population Sampling

We sampled wild populations of Drosophilids at five locations across the native range of *Z. indianus* in sub-Saharan Africa and three locations in the invasive range in North America and Hawaii (fig. 1) using traps baited with bananas.

### Genome Assembly and Annotation

To perform population genomic analyses, we first generated genome assemblies de novo for each of the seven *Zaprionus* species included in this study using data generated from Illumina and Nanopore sequencers. We used the BUSCO annotation pipeline (Waterhouse et al. 2018) to assess assembly quality and quantify the presence of—and generate annotations for—2,799 benchmarking universal single-copy orthologs that have been curated in 25 different species of Diptera. Finally, we generated gene annotations using RNA sequence data and the MAKER annotation pipeline (Campbell et al. 2014) for six of the seven de novo genome assemblies (we did not collect RNA-seq data for *Z. inermis*). Our sequencing, assembly, and annotation approaches resulted in genome assemblies with scaffold N50s between 336 kb and 2.45 Mb, complete single-copy BUSCO annotations of 90.7–97.4%, and 9,275–11,071 annotated transcripts (supplementary table S2, Supplementary Material online).

### Population Resequencing and Genotyping

To quantify genetic diversity within populations of *Zaprionus*, we generated resequence data from 93 individuals across 16 populations and 7 species (minimum *N* = 3; maximum *N* = 11; supplementary table S3, Supplementary Material online). We mapped raw sequence reads using the BWA*mem* algorithm (v0.7.15), sorted, and filtered mapped reads using SAMTOOLS (v1.4), marked duplicates using the PICARD*MarkDuplicates* tool (v2.2.4), and realigned around indels using GATK’s *RealignerTargetCreator* and *IndelRealigner* tools (v3.8; McKenna et al. 2010). We estimated genotypes for each individual using GATK and hard-filtered sites genotyped in fewer than two individuals (VCFtools [v0.1.15] option “–max-missing 0.5”). To facilitate comparisons across populations where we sampled different numbers of individuals, joint genotyping and filtering were carried out on randomly selected groups of four individuals (eight chromosomes) per population, except for the population of *Z. africanus* sampled from São Tomé, where we only sampled three individuals.

### Estimating Genetic Diversity

Population genetic metrics of genetic diversity were computed using VCFtools with coverage-masked sites excluded using the “–exclude-positions” filter option. Because *π*_SNP_ and *S* were highly correlated in all populations (*r *>* *0.963), we focus primarily on *S*: the number of sites with segregating variation within a given 5-kb window.

### Comparing Genetic Diversity within and outside of Gene Annotations

We used GLMs with Poisson distributed error (*glm*() function in R) to model the number of segregating sites (*S*) within a genomic window as a function of the position of that window relative to a gene annotation. For populations of *Z. indianus*, we were also interested in whether aspects of biological invasion had a different effect on levels of genetic diversity depending on the proximity of a genomic region to a gene. We therefore used a GLM to test the interaction between gene region type (i.e., overlapping, adjacent, or distant) and invasion status (i.e., invasive population of *Z. indianus*, native population of *Z. indianus*, or population of noninvasive species of *Zaprionus*) on median levels of genetic diversity across genomic windows.

### Estimating Recombination Rate and Its Effect on Genetic Diversity

To estimate fine-scale population recombination rates (*ρ*_rec_ = 2*Nr*) across the genome, we used the maximum likelihood method implemented in LDhelmet (v1.10; Chan et al. 2012). For analyses involving recombination rate, we focused on populations of *Z. indianus* because we had the largest sample size of this species from a single region (Senegal: *N* = 14 individuals; 28 haplotypes), which allowed us to use haplotype information to estimate recombination rates across the genome. All analyses below are conducted on mean recombination rates within 5,000-bp genomic windows that were generated from median posterior estimates provided by LDhelmet.

We tested for a relationship between recombination rate and gene density across the 40 largest scaffolds of the *Z. indianus* genome assembly (12,080 windows) using Spearman’s rank correlation tests. We found no evidence for a relationship between recombination rate and gene density—either across all windows (Spearman’s *ρ* = −0.014; *P *=* *0.12) or windows containing at least one gene (*N* = 4,406 windows; Spearman’s *ρ*  =  0.023; *P *=* *0.12). We therefore conducted independent tests for relationships between genetic diversity and recombination rate and genetic diversity within and around gene annotations.

### Measuring the Correlation in Genetic Diversity between Species

To facilitate interspecific comparisons, we analyzed genomic regions that spanned the first and last exon of an annotated BUSCO ortholog. In total, we quantified the correlation in diversity (or lack thereof) across 2,714 genomic windows spanning BUSCO orthologs (hereafter “BUSCO windows”) annotated across all seven species’ genome assemblies. Correlation coefficients were estimated using the *rcorr()* function from the Hmisc R package. To test whether comparing BUSCO windows produced correlations greater than expected by chance, we performed randomization tests where we randomly selected 2,714 genomic windows (100 iterations) and computed all pairwise Spearman’s rank correlation coefficients for those windows across species.

The genomic landscape of diversity may diverge between species with increasing phylogenetic distance due to aspects of genome evolution. To quantify whether this would affect interspecific comparisons of genetic diversity, we computed phylogenetic distances between each species using a concatenated alignment of 1,709 BUSCOs shared across all species genome assemblies and representing 4,616,644 sites. Genetic distances were computed from the identity matrix of the aligned BUSCOs using the *dist.alignment()* function from the seqinr R library. We then tested whether the correlation in *S* across the genome of two species was related to their genetic distance (Spearman’s Mantel test; *mantel()* function in the ecodist R library). We also calculated Spearman’s rank correlations for *S* and Tajima’s *D* between populations of *Z. indianus* (all pairwise comparisons) using diversity estimates across all 5-kb genomic window.

### Estimating Thermal Performance Curves

To quantify differences in performance across populations, we fit a model that jointly describes the thermal performance curve to data from all populations using a hierarchical Bayesian framework (Tittes et al. 2019). This model provides estimates for the maximum height of a population’s performance curve, two shape parameters allowing for asymmetry in the performance curve, and the minimum and maximum temperatures where performance falls to 0 (*T*_min_ and *T*_max_ parameters, respectively). The height (i.e., stretch as defined in Tittes et al. 2019) reflects the maximal fitness/performance of a population. We also derived three additional parameters from the five model-estimated parameters to summarize aspects of fitness and the thermal niche: the temperature at which a population displays maximal performance (*T*_optimum_), the total area under the estimated performance curve (*A*_c_), and the breadth of the curve between the lower 25% and the upper 25% of the curve (*B*_50_). We compared performance curves between species by generating posterior draws of parameter estimates. Two populations were considered to differ with respect to parameters describing their thermal performance curve when a given parameter estimate had a probability >0.95 of being different between the two populations. 

## Supplementary Material

Supplementary data are available at *Molecular Biology and Evolution* online.

## Supplementary Material

msaa050_Supplementary_Data

## Notes

Raw sequences at the NCBI short-read archive under BioProject PRJNA604690. Genome assemblies, annotations, and phenotypic data at Dryad: https://doi.org/10.5061/dryad.866t1g1n3.
