# Supplementary material for: Genetic Diversity and Thermal Performance in Invasive and Native Populations of African Fig Flies
Source: Mol Biol Evol. 2020 Feb 28;37(7):1893–906. doi: 10.1093/molbev/msaa050 (PMC7306694; doi:10.1093/molbev/msaa050)
Supplement: msaa050_Supplementary_Data [file msaa050_supplementary_data.pdf]

Supplementary Material for:

Genetic diversity and thermal performance in invasive and native populations of African fig flies

Aaron A. Comeault[1,2], Jeremy Wang[3], Silas Tittes[4], Kristin Isbell[2], Spencer Ingley[5], Allen H. Hurlbert[2], Daniel R. Matute[2]

[1]School of Natural Sciences, Bangor University, Bangor, Gwynedd, LL57 2DGA, UK

[2]Department of Biology, University of North Carolina, Chapel Hill, NC, 27599, USA

[3]Department of Genetics, University of North Carolina, Chapel Hill, NC, 27599, USA

[4]Department of Evolution and Ecology, University of California, Davis, CA, 95616, USA

[5]Faculty of Sciences, Brigham Young University, Hawaii, Laie, 96762 USA

Corresponding author: a.comeault@bangor.ac.uk

This file contains:

1. Supplementary Methods; pp. 2-9
2. Supplementary References; p. 10
3. Supplementary Tables S1 – S7; pp. 11-17
4. Supplementary Figures S1 – S6; pp. 18-23

1. Supplementary Methods

1.1 Sampling procedure

To assess patterns of genetic diversity across populations of *Z. indianus* we sampled populations from Kenya, Zambia, São Tomé, and Senegal (both forest/savannah outside of Niokolo-Koba National Park and coastal desert in the northwest of the country) in their native Africa, and Hawaii (Oahu), Tennessee (Nashville), and North Carolina (Chapel Hill) in their invasive range. Across African locations we also sampled other *Zaprionus* species, including two populations of *Z. africanus* (São Tomé and Kenya), two populations of *Z. tuberculatus* (São Tomé and the Senegal-desert site), and one population each of *Z. inermis*, *Z. tsacasi*, *Z. taronus*, and *Z. nigranus* (São Tomé). Live flies were aspirated directly from traps and within one hour of collection, anesthetised with flynap (triethylamine in alcohols; Carolina Biological Supply) and identified under light microscopes. Up to 50 individual females from the genus *Zaprionus* (per sample location) were moved into vials containing hydrated instant drosophila media to establish isofemale lines. Males and excess females were preserved in 100% ethanol.

1.2 Genome assembly

For each species, we extracted genomic DNA from pools of 5 to 30 male flies from a single isofemale line and sequenced those extractions using Illumina and Oxford Nanopore (ONT) sequencers or, for a subset of species, ONT sequencers only (Table S1). For species with both Illumina and ONT data, we generated initial assemblies with `SPAdes` v3.12.0 (Bankevich et al. 2012). Assembled contigs were processed by `Redundans` v0.13c (Pryszcz and Gabaldón 2016) to remove residual redundant haplotypes. Nanopore reads were corrected using FMLRC (Wang et al. 2018) and used to scaffold assembled contigs using LINKS v1.8.5 (Warren et al. 2015) with the recommended iterative approach (https://github.com/bcgsc/LINKS). Resulting scaffolds were corrected, and consensus sequences were generated, using `Racon` v1.3.2 (Vaser, Sović, Nagarajan, & Šikić, 2017) and `Pilon` v2.11 (Walker et al. 2014). For species with

nanopore-only data, we mapped reads in a pairwise fashion using Minimap2 v2.15-r905 (Li 2018: 2) and assembled with Miniasm v0.3-r179 (Li 2016). We then corrected and generated consensus genome sequences with four iterations of Racon v1.3.2 (Vaser et al. 2017) followed by Medaka v0.6.2 (https://github.com/nanoporetech/medaka), respectively.

1.3 Annotation

We isolated total RNA using a standard TRIzol protocol from sex-specific groups of one day old adult flies (2 to 5 flies per extraction) that were flash frozen in liquid nitrogen. Stranded RNA-seq libraries were then constructed for each pool and sequencing was carried out on two lanes of an Illumina HiSeq 2500, run in rapid mode with 2 x 150 cycles. This sequencing approach generated between 21 and 32 million reads per extraction. Library construction and sequencing was carried out at the University of North Carolina Medical School's High-Throughput Sequencing Facility. We assembled a transcriptome for *Z. africanus* (1m, 2f pools), *Z. tuberculatus* (2f pools), *Z. nigranus* (2m, 2f pools), *Z. indianus* (2m, 2f pools), and *Z. tsacasi* (1m, 1f pools) using Trinity (Grabherr et al. 2011; Haas et al. 2013) run with default parameters. We then used MAKER (v3.01.02; (Holt and Yandell 2011; Campbell et al. 2014)) to annotate each genome, including RepeatMasker (v4.07; (Smit and Green 2013)) and est2genome to directly predict genes from assembled transcripts. Functional annotations were predicted using BLASTP (v2.7.1) against Swiss-Prot with an e-value cutoff of 0.000001.

1.4 Resequencing and genotyping

We extracted genomic DNA from either individual wild-caught flies or from a single offspring of a wild-caught female (i.e. first generation offspring) using Genetra Puregene Tissue Kits (Qiagen, Valencia, CA, USA), constructed barcoded libraries for sequencing using KAPA HyperPrep kits (Roche Sequencing, Pleasanton, CA) with a target fragment size of 500 bp, and sequenced in pools of 10 to 20 libraries per lane on either Illumina HiSeq 2500 or 4000 machines, generating either 2×125bp or 2×150bp reads,

respectively. Library preparation and sequencing was done at the University of North Carolina (UNC) School of Medicine's high-throughput sequencing facility.

Raw sequence data was initially parsed and barcodes were removed by the UNC High-throughput sequencing facility. We then mapped parsed reads to each individual's respective reference genome using the BWA *mem* algorithm (v0.7.15). We sorted and filtered mapped reads using SAMTOOLS (v1.4), marked duplicates using the PICARD *MarkDuplicates* tool (v2.2.4), and realigned around indels using GATK's *RealignerTargetCreator* and *IndelRealigner* tools (v3.8; (McKenna et al. 2010)). Processed alignment files (.bam format) were generated separately for each individual using this pipeline.

We estimated genotypes for each individual using GATK's *HaplotypeCaller* tool with options "--emitRefConfidence GVCF", "--minReadsPerAlignmentStart 4", "--standard_min_confidence_threshold_for_calling 8.0", and "--minPruning 4". We then performed joint genotyping using GATK's *GenotypeGVCFs* tool. We filtered SNPs using GATK's *VariantFiltration* tool with option "--filterExpression "QD < 2.0 || FS > 60.0 || SOR > 3.0 || MQ < 40.0 || MQRankSum < -12.5 || ReadPosRankSum < -8.0"" and hard-filtered sites genotyped in fewer than two individuals (VCFtools (v0.1.15) option "--max-missing 0.5"). To facilitate comparisons across populations where we sampled different numbers of individuals, joint genotyping and filtering was carried out on randomly selected groups of four individuals (8 chromosomes) per population, except for the population of *Z. africanus* sampled from São Tomé, where we only sampled three individuals. Lastly, for each species, we masked sites in the genome if coverage was greater than twice, or less than half, the average coverage observed across all sequenced individuals of that species.

1.5 Differentiation among populations of Z. indianus

We estimated differentiation among populations of *Z. indianus* using principal component analysis, population assignment, and genomic window analyses. Principal component analysis (PCA) and population assignment were carried out using the *admix* method implemented in PCAngsd (Meisner and Albrechtsen 2018). These analyses were

carried out on genotype likelihoods estimated using the GATK method implemented in ANGSD (McKenna et al. 2010; Korneliussen et al. 2014). To explore genome-wide differentiation between populations we estimated F_{ST} in 5 kb non-overlapping genomic windows using VCFtools (v0.1.15). We first jointly genotyped all *Z. indianus* samples using GATK's *GenotypeGVCF*s tool and applied filtering as described above (see section 1.4). The resulting filtered set of variants was used as input for the window analysis conducted with VCFtools.

1.6 Estimating genetic diversity

We estimated kinship, using filtered genotypes, for all within-population pairwise comparisons using the KING method (Manichaikul et al. 2010) as implemented in the VCFtools "--relatedness2" tool and estimated the inbreeding coefficient (F) for each individual based on filtered genotypes using the VCFtools "--het" tool. We excluded coverage-masked sites using the "--exclude-positions" filter option. Because scaffolds in our assemblies belonging to the sex chromosomes have not been identified, we restricted our analysis of inbreeding to include only females, because homozygous genotype calls for males on the X chromosome would inflate estimates of inbreeding. In total, we estimated F for 7 females from the invasive range and 12 females from the native range of Z. indianus.

Population genetic metrics of genetic diversity were computed using VCFtools with coverage-masked sites excluded using the "--exclude-positions" filter option. Because πSNP and S were highly correlated in all populations ($r > 0.963$), we focus primarily on S: the number of sites with segregating variation within a given 5 Kb window. We summarize estimates of genetic diversity within each population as median, 5% empirical quantile, and 95% empirical quantile values (Table S4).

We explored the effect of being located in or around genes on levels of genetic diversity by first comparing genetic diversity across all genomic windows to diversity in windows that overlapped an annotated BUSCO gene (Waterhouse et al. 2018). We include this category of distinct annotations because these genes (2,799 total) have been curated as single-copy orthologs in 25 dipteran species and we were able to annotate a

high percentage (minimum 90.7%, maximum 97.3%) as being present in complete single-copies in the de novo assemblies we generated for this study (Table S2). Comparing diversity within these “BUSCO windows” allows for less biased comparisons between species because these windows should be less affected by aspects of genome evolution such as changes in gene copy number.

To test whether genomic regions that overlapped with gene annotations differed in levels of genetic diversity compared to regions away from genes, we used generalized linear models (GLMs) with poisson distributed error (glm() function in R) to model the number of segregating sites (S) within a genomic window as a function of the position of that window relative to a gene annotation. We classified genomic windows as overlapping, adjacent to (within 5,000 bp), or distant from (> 5,000 bp) the nearest gene annotation. We carried out this analysis separately for each population for which we generated annotations for their species' respective genome assembly (N = 15). For populations of *Z. indianus*, we were also interested in whether aspects of biological invasion had a different effect on levels of genetic diversity depending on the proximity of a genomic region to a gene. We therefore used a GLM to test the interaction between gene region type (i.e. overlapping, adjacent, or distant) and invasion status (i.e. invasive population of *Z. indianus*, native population of *Z. indianus*, or population of non-invasive species of *Zaprionus*) on median levels of genetic diversity across genomic windows.

1.7 Estimating recombination rates across the Z. indianus *genome and its effect on genetic diversity*

We estimated population recombination rates ($\rho_{rec} = 2Nr$) across the *Z. indianus* genome using the maximum likelihood method implemented in `LDhelmet` (v1.10; (Chan et al. 2012)). Before running `LDhelmet`, we generated phased haplotypes for the 14 *Z. indianus* sampled from Senegal (generating 28 phased haplotypes) using read-aware phasing (`Shapeit v2.837`; (Delaneau et al. 2013)). Phasing was carried out for the 40 largest scaffolds of the *Z. indianus* assembly, totalling 61.2 Mb of sequence or ~42% of the genome. We then ran `LDhelmet` on the phased data by first generating haplotype configuration files for each individual using the “find_confs” script, specifying a window

size of 50 SNPs. We then computed lookup tables using the "table_gen" script, specifying a population-scaled mutation rate of θ = 0.034 (Watterson's θ; estimated from the data using `ANGSD`), and a grid of recombination rate values of [0.0 0.1 10.0 1.0 100.0]. We estimated Padé coefficients, specifying θ = 0.034 and 11 replicates. Finally, we estimated recombination rates, running `LDhelmet`'s rjMCMC algorithm with a block penalty of 10 and a burn-in of 100,000 MCMC iterations followed by 1,000,000 MCMC iterations and extracted the mean recombination rate estimate between each pair of SNPs using `LDhelmet`'s "post_to_text" script. To summarize variation in recombination rate across the genome, we first removed unrealistically high estimates of ρ_{rec} (i.e. $\rho_{rec} > 1$, corresponding to a recombination rate greater than ~ $15*10^{-8}$) and then calculated the mean recombination rate in 5000 bp windows across the genome from median estimates provided by `LDhelmet`.

We tested for a correlation between genetic diversity (S) and recombination rate, for each population of *Z. indianus*, by calculating Spearman's ρ using the cor.test() function in R. We calculated the correlation between median recombination rate and the mean difference in S between invasive and native populations of *Z. indianus* across genomic windows. Because recombination rate was positively correlated with diversity and the number of SNPs in a genomic window affects the magnitude of change in genetic diversity that is possible, we restricted this analysis to windows with a mean number of SNPs in the across populations of *Z. indianus* in their native range between 150 and 300. To further account for differences in diversity across windows as a function of local recombination rate we scaled the observed difference in mean S between the invasive and native ranges of *Z. indianus* by the mean number of SNPs within genomic windows binned into five recombination rate quantiles. Our rationale for this approach was to test whether the proportional reduction in diversity in invasive populations of *Z. indianus* was greater for regions of the genome with low recombination rates, as would be expected if selection was acting to drive the genetic diversity lower in invasive populations.

1.8 Measuring thermal performance

We measured adult-to-adult performance under four different temperatures and a 10:14 hour night:day light cycle. Temperatures were (night:day) 11°C:16°C, 16°C:21°C, 21°C:26°C, and 26°C:31°C, resulting in mean hourly temperatures of 13.9, 18.9, 23.9, and 28.9°C, respectively. To initiate the experiment, individuals were collected as one to three-day old adults, briefly anesthetized with CO_2, and placed as individual pairs into vials containing standard cornmeal agar medium. Each pair was then allowed to recover from anesthesia for 12 to 24 hours at room temperature before being randomly assigned to one of the four temperature treatments. Each pair of flies was then allowed to lay eggs for 7 to 9 days, after which they were removed from the vials and a dampened kimwipe was added to each vial as a pupation site for the larvae. We then counted the total number of offspring that successfully enclosed within each vial. We measured adult-to-adult performance in this way for a total of 900 pairs, with an average of 9 pairs per temperature per species. Temperature and light was controlled using Percival incubators (model DR-36VL). Relative humidity within the incubators was negatively correlated with temperature, but was maintained between 80% and 50%.

We modified the original model presented in (Tittes et al. 2019) to account for higher variability in reproduction and survival in our dataset compared to the data on which the model was originally developed. We modeled the data as a mixture of a Gaussian probability density that described thermal performance and a Bernoulli probability mass that described excess zeros caused by mortality and failure of pairs to reproduce, which we will subsequently refer to as "mortality" for simplicity. We assumed the probability of mortality was inversely proportional to the mean thermal performance, such that more zeros are expected to occur near the thermal tolerance limits. The Bayesian p-value for the model was 0.77, indicating an adequate goodness-of-fit of the model to the data. Other modeling details including values chosen for priors remained the same as in Tittes et al. (2019). We have posted the Stan code that provides a precise description of the model at: https://github.com/silastittes/performr/tree/zin.

In the main text, we report on parameter estimates of thermal performance minimum (T_{min} == x_min), thermal maximum (T_{max} == x_max), thermal optimum ($T_{optimum}$ == maxima), maximum realized fitness (max. fitness == stretch), and a measure of thermal niche breadth (B_{50}), each derived from the estimated thermal performance

curves. Each of these parameters, except for B_{50}, is described in Tittes et al. (2019). We estimated B_{50} as the difference in temperature values that captured the central 50% of the curve area, and was calculated as the difference between

critical = [(1 - ((1 - X_CRITICAL) ^ (1/shape_2)))^(1/shape_1)] * (x_max - x_min) + x_min, where X_CRITICAL was chosen to be 0.75 0.25, respectively.

2. Supplementary References

Bankevich A, Nurk S, Antipov D, Gurevich AA, Dvorkin M, Kulikov AS, Lesin VM, Nikolenko SI, Pham S, Prjibelski AD, et al. 2012. SPAdes: A New Genome Assembly Algorithm and Its Applications to Single-Cell Sequencing. J. Comput. Biol. 19:455–477.

Campbell MS, Holt C, Moore B, Yandell M. 2014. Genome Annotation and Curation Using MAKER and MAKER-P. Curr. Protoc. Bioinforma. 48:4.11.1-39.

Chan AH, Jenkins PA, Song YS. 2012. Genome-Wide Fine-Scale Recombination Rate Variation in Drosophila melanogaster. PLOS Genet. 8:e1003090.

Grabherr MG, Haas BJ, Yassour M, Levin JZ, Thompson DA, Amit I, Adiconis X, Fan L, Raychowdhury R, Zeng Q, et al. 2011. Full-length transcriptome assembly from RNA-Seq data without a reference genome. Nat. Biotechnol. 29:644–652.

Haas BJ, Papanicolaou A, Yassour M, Grabherr M, Blood PD, Bowden J, Couger MB, Eccles D, Li B, Lieber M, et al. 2013. De novo transcript sequence reconstruction from RNA-Seq: reference generation and analysis with Trinity. Nat. Protoc. [Internet] 8. Available from: https://www.ncbi.nlm.nih.gov/pmc/articles/PMC3875132/

Holt C, Yandell M. 2011. MAKER2: an annotation pipeline and genome-database management tool for second-generation genome projects. BMC Bioinformatics 12:1–14.

Korneliussen TS, Albrechtsen A, Nielsen R. 2014. ANGSD: Analysis of Next Generation Sequencing Data. BMC Bioinformatics 15:356.

Li H. 2016. Minimap and miniasm: fast mapping and de novo assembly for noisy long sequences. Bioinformatics 32:2103–2110.

Li H. 2018. Minimap2: pairwise alignment for nucleotide sequences. Bioinformatics 34:3094–3100.

McKenna A, Hanna M, Banks E, Sivachenko A, Cibulskis K, Kernytsky A, Garimella K, Altshuler D, Gabriel S, Daly M, DePristo MA. 2010. The genome analysis toolkit: A MapReduce framework for anlayzing next-generation DNA sequencing data. Genome Res. 20:254-260

Meisner J, Albrechtsen A. 2018. Inferring Population Structure and Admixture Proportions in Low-Depth NGS Data. Genetics 210:719–731.

Pryszcz LP, Gabaldón T. 2016. Redundans: an assembly pipeline for highly heterozygous genomes. Nucleic Acids Res. 44:e113–e113.

Smit A, Green P. 2013. RepeatMasker Open-4.0. Available from: http://www.repeatmasker.org

Tittes SB, Walker JF, Torres-Martínez L, Emery NC. 2019. Grow Where You Thrive, or Where Only You Can Survive? An Analysis of Performance Curve Evolution in a Clade with Diverse Habitat Affinities. Am. Nat. 193:530–544.

Vaser R, Sović I, Nagarajan N, Šikić M. 2017. Fast and accurate de novo genome assembly from long uncorrected reads. Genome Res. 27:737–746.

Walker BJ, Abeel T, Shea T, Priest M, Abouelliel A, Sakthikumar S, Cuomo CA, Zeng Q, Wortman J, Young SK, et al. 2014. Pilon: An Integrated Tool for Comprehensive Microbial Variant Detection and Genome Assembly Improvement. PLOS ONE 9:e112963.

Wang JR, Holt J, McMillan L, Jones CD. 2018. FMLRC: Hybrid long read error correction using an FM-index. BMC Bioinformatics 19:50.

Warren RL, Yang C, Vandervalk BP, Behsaz B, Lagman A, Jones SJM, Birol I. 2015. LINKS: Scalable, alignment-free scaffolding of draft genomes with long reads. GigaScience [Internet] 4. Available from: https://academic.oup.com/gigascience/article/4/1/s13742-015-0076-3/2707579

Table S1. Summary of the data used to generate each of the seven draft genome assemblies reported in the main text. The amount of sequence data generated for each species and each sequencing technology is given in billions of base pairs (Gbp). For nanopore sequencing we also report the number of reads in millions (M) and the mean read length in base pairs.

species	Collection location	Illumina read type	Illumina amount	Nanopore reads (N)	Nanopore amount	Nanopore mean read length
Z. indianus	Florida	MiSeq 250 x 2	8.6 Gbp	1.7M	4.2 Gbp	2471
Z. africanus	Sao Tome	n/a	n/a	1.7M	7.4 Gbp	4259
Z. nigranus	Sao Tome	MiSeq 300 x 2	11.1 Gbp	1.6M	1.9 Gbp	1250
Z. taronus	Sao Tome	n/a	n/a	2.5M	10.0 Gbp	3900
Z. inermis	Sao Tome	n/a	n/a	3.1M	10.6 Gbp	3400
*Z. tuberculatus**	Zambia	n/a	n/a	n/a	n/a	n/a
Z. tsacasi	Sao Tome	HiSeq 2500 150 x 2	14.9 Gbp	.945M	2.5 Gbp	2646

* The *Z. tuberculatus* assembly was generated by Dovetail genomics with their proprietary Chicago libraries, a Hi-C library, and Illumina sequence data.

Table S2. Summary statistics for each of the seven draft genome assemblies reported in the main text. BUSCO annotation report is based on a total of 2799 single copy orthologs curated in 25 genomes of different species of Diptera. Annoations were generated using RNA-seq data, Trinity, and the MAKER annotation pipeline (see Supplementary Methods and the Main Text).

species	Assembly size (Mbp)	# contigs	N50 (bp)	% complete BUSCO	% complete single-copy BUSCO	% duplicated BUSCO	# annotated transcripts
Z. indianus	145.7	649	773,890	96.6	96.1	0.5	10,013
Z. africanus	167.6	689	1,499,604	95	94.1	0.9	10,424
Z. nigranus	142.9	2553	776,169	97.8	97.3	0.5	9,769
Z. taronus	187.9	536	2,214,536	95.2	93.7	1.5	9,275
Z. inermis	165.5	572	2,453,702	94.9	94	0.9	n/a
Z. tuberculatus	176.2	880	25,350,852	93.2	90.7	2.5	11,071
Z. tsacasi	150.1	1269	335,836	96.3	95.7	0.6	10,408

Table S3. Populations and sample sizes for which whole genome resequencing data was generated to estimate genetic diversity. Sample sizes are reported as the number of chromosomes sampled from each population (i.e. 2 x the number of individuals sampled).

species	location	2N
Z. indianus	Hawaii	8
Z. indianus	North Carolina	12
Z. indianus	Tennessee	8
Z. indianus	Sao Tome	12
Z. indianus	Senegal (forest)	14
Z. indianus	Senegal (desert)	14
Z. indianus	Kenya	14
Z. indianus	Zambia	12
Z. africanus	Sao Tome	6
Z. africanus	Kenya	10
Z. nigranus	Sao Tome	10
Z. taronus	Sao Tome	22
Z. inermis	Sao Tome	8
Z. tuberculatus	Senegal	14
Z. tuberculatus	Sao Tome	14
Z. tsacasi	Sao Tome	8

Table S4. Summary of genetic diversity within each population. Population identifiers are in the format species-POPULATION-replicate, where species is abbreviated as "z" followed by the first three letters of the species name and population is the abbreviation of the collection location. Summary statistics were calculated from groups of four individuals and in populations where more than four individuals were sampled, multiple subsamples (replicates) were analyzed. Mean, 5% empirical quantile (5%), and 95% empirical quantile (95%) for nucleotide diversity (*pi*), the number of segregating sites (*S*), and Tajima's *D* (T. *D*) calculated in 5 kb windows across the genome are reported.

population	*pi* median	*pi* (5%)	*pi* (95%)	*S* median	*S* (5%)	*S* (95%)	T. *D* median	T. *D* (5%)	T. *D* (95%)
zafr-KEN-1	0.0169	0.0011	0.0276	231	3	366.2	-0.66	-1.10	0.11
zafr-KEN-2	0.0173	0.0011	0.0279	235	4	371	-0.68	-1.13	0.09
zafr-ST-1	0.0188	0.0010	0.0304	205	3	332	-0.42	-0.93	0.49
zind-HI-1	0.0110	0.0006	0.0206	120	0	231	0.51	-1.28	2.09
zind-KEN-1	0.0171	0.0030	0.0274	219	37	339	-0.54	-1.05	0.17
zind-KEN-2	0.0175	0.0029	0.0279	230	36	349	-0.67	-1.15	0.02
zind-NC-1	0.0138	0.0016	0.0241	160	10	281	0.17	-1.02	1.71
zind-NC-2	0.0137	0.0014	0.0241	159	7	281	0.14	-1.18	1.64
zind-SEN desert-1	0.0174	0.0021	0.0275	230	28	347	-0.71	-1.18	-0.17
zind-SEN desert-2	0.0175	0.0022	0.0276	231	28	348	-0.70	-1.17	-0.14
zind-SEN forest-1	0.0176	0.0021	0.0278	232	28	350	-0.71	-1.18	-0.17
zind-SEN forest-2	0.0176	0.0021	0.0279	233	28	350	-0.72	-1.18	-0.15
zind-ST-1	0.0161	0.0018	0.0271	206	21	336	-0.48	-1.05	0.58
zind-ST-2	0.0161	0.0018	0.0271	206	21	335	-0.48	-1.04	0.54
zind-TN-1	0.0136	0.0015	0.0241	156	4	280.65	0.22	-0.92	1.93
zind-ZAM-1	0.0168	0.0026	0.0276	222	32	349	-0.66	-1.16	0.45
zind-ZAM-2	0.0172	0.0027	0.0278	229	33	350	-0.71	-1.22	-0.12
zine-ST-1	0.0031	0.0001	0.0084	35	0	92	0.52	-1.74	2.07
znig-ST-1	0.0018	0.0005	0.0045	23	6	55	0.16	-0.90	1.29
znig-ST-2	0.0019	0.0005	0.0045	23	6	55	0.12	-0.92	1.14
ztar-ST-1	0.0095	0.0003	0.0219	111	0	260	-0.34	-1.17	0.42
ztar-ST-2	0.0092	0.0003	0.0216	107	0	257	-0.30	-1.15	0.43

ztar-ST-3	0.0090	0.0003	0.0216	100	0	256	-0.20	-1.06	0.79
ztsa-ST-1	0.0115	0.0007	0.0214	143	5	257	-0.21	-0.88	0.53
ztub-SEN-1	0.0100	0.0003	0.0191	133	2	247	-0.41	-1.03	0.42
ztub-SEN-2	0.0093	0.0003	0.0179	126	1	239	-0.33	-0.89	0.61
ztub-ST-1	0.0122	0.0003	0.0223	152	1	272	-0.37	-0.89	0.52
ztub-ST-2	0.0122	0.0003	0.0224	152	2	273	-0.39	-0.97	0.39

Table S5. Summary of genetic diversity within each population, as reported in Table S4, but restricted to genomic windows overlapping an annotated BUSCO gene. Only one replicate of four randomly selected individuals was run for each population, as results in Table S4 indicate that there was not a large variance between estimates generated from different subsamples of four individuals.

population	*pi* (median)	*pi* (5%)	*pi* (95%)	*S* (median)	*S* (5%)	*S* (95%)	T. *D* (median)	T. *D* (5%)	T. *D* (95%)
zind-HI	0.0099	0.0018	0.0203	109	0	231	0.48	-1.26	2.10
zind-NC	0.0123	0.0031	0.0241	147	28.85	281.15	0.15	-0.98	1.69
zind-TN	0.0121	0.0029	0.0241	142	20	281	0.24	-0.77	1.96
zind-ST	0.0147	0.0032	0.0276	193	43	342	-0.51	-1.09	0.36
zind-SEN desert	0.0156	0.0052	0.0278	213	79	351	-0.72	-1.21	-0.28
zind-SEN forest	0.0157	0.0052	0.0282	215	80	356	-0.72	-1.21	-0.28
zind-KEN	0.0153	0.0053	0.0275	203	74.7	341.15	-0.55	-1.06	0.11
zind-ZAM	0.0152	0.0051	0.0280	206	71.85	353.15	-0.67	-1.22	0.15
zafr-ST	0.0196	0.0056	0.0319	221	67	354	-0.49	-0.90	0.11
zafr-KEN	0.0178	0.0050	0.0296	251	81	394.3	-0.71	-1.13	-0.26
ztub-ST	0.0124	0.0046	0.0211	160	61	263	-0.40	-0.84	0.14
ztub-SEN	0.0109	0.0037	0.0192	150	50	252	-0.45	-0.87	0.23
zinerm-ST	0.0029	0.0001	0.0070	33	1	77	0.53	-1.80	2.08
ztsac-ST	0.0095	0.0017	0.0206	121	24	247	-0.21	-0.97	0.39
znig-ST	0.0013	0.0004	0.0036	17	4	44	0.09	-1.03	1.19
ztar-ST	0.0064	0.0011	0.0194	85	17	237	-0.36	-1.29	0.35

Table S6. Mean number of segregating sites (*S*) across 5 kb genomic windows grouped by their location relative to an annotated gene. Windows were classified as overlapping a gene, within 5 kb, but not overlapping (adjacent), or further than 5kb from a gene (distant). The last two columns show relative amounts of genetic diversity contained within windows that overlapped an annotated gene and either adjacent or distant genomic windows.

population	overlapping	adjacent	distant	overlapping/adjacent	overlapping/distant
zind-HI	129	134	133	0.9626866	0.9699248
zind-NC	168	176	172	0.9545455	0.9767442
zind-TN	166	172	167	0.9651163	0.994012
zind-ST	221	230	224	0.9608696	0.9866071
zind-SENdesert	246	257	256	0.9571984	0.9609375
zind-SENforest	249	260	258	0.9576923	0.9651163
zind-KEN	233	242	243	0.9628099	0.9588477
zind-ZAM	238	248	243	0.9596774	0.9794239
zafr-ST	237	230	214	1.0304348	1.1074766
zafr-KEN	274	264	248	1.0378788	1.1048387
ztub-ST	161	167	173	0.9640719	0.9306358
ztub-SEN	146	145	145	1.0068966	1.0068966
ztsac-ST	138	153	168	0.9019608	0.8214286
znig-ST	20	24	27	0.8333333	0.7407407
ztar-ST	102	130	152	0.7846154	0.6710526

Table S7. Pairwise sequence differences (percent differences) between the seven *Zaprionus* species analyzed in the main text. Genetic distances are derived from the 1709 BUSCOs used to generate the phylogeny shown in Figure 5a of the main text.

	africanus	*indianus*	*inermis*	*nigranus*	*taronus*	*tsacasi*	*tuberculatus*
africanus	0.0						
indianus	4.58	0.0					
inermis	13.69	13.48	0.0				
nigranus	6.29	5.98	13.15	0.0			
taronus	6.78	6.58	13.53	4.30	0.0		
tsacasi	13.43	13.20	8.87	12.87	13.27	0.0	
tuberculatus	13.54	13.34	9.05	12.97	13.37	4.38	0.0

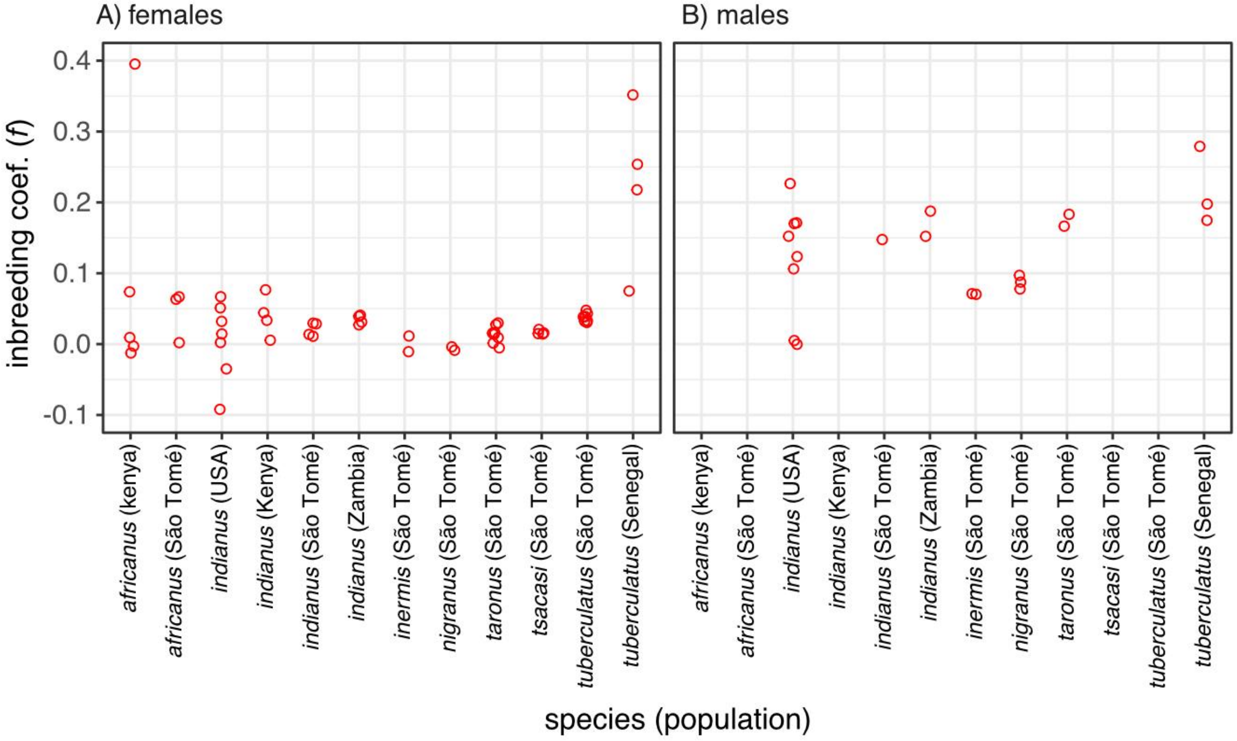

Figure S1. Estimated inbreeding coefficient (*f*) for all sequenced individuals with known sex at time of sequencing (A: females; B: males). Inbreeding coefficients were estimated using the KING method as implemented in VCFTools (see Main Text for details).

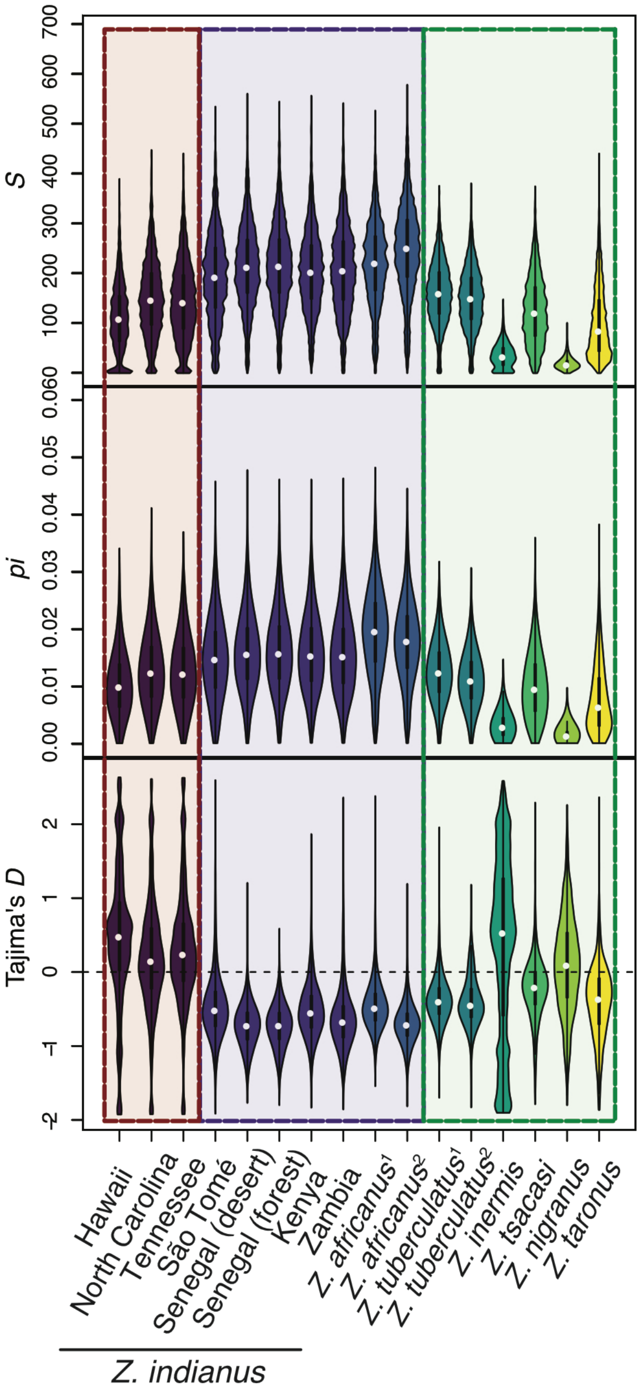

Figure S2. Estimates of genetic diversity summarized across 5 kb genomic windows that overlap with an annotated BUSCO. Shaded boxes group populations as invasive *Z. indianus* (three leftmost violins), native *Z. indianus* and *Z. africanus* (seven central violins), and other species (six rightmost violins). See Figure 2 in the main text for results across the entire genome. *Z. africanus*[1] and *Z. tuberculatus*[1] were sampled from Sao Tome, *Z. africanus*[2] from Kenya, and *Z. tuberculatus*[2] from Senegal (forest site).

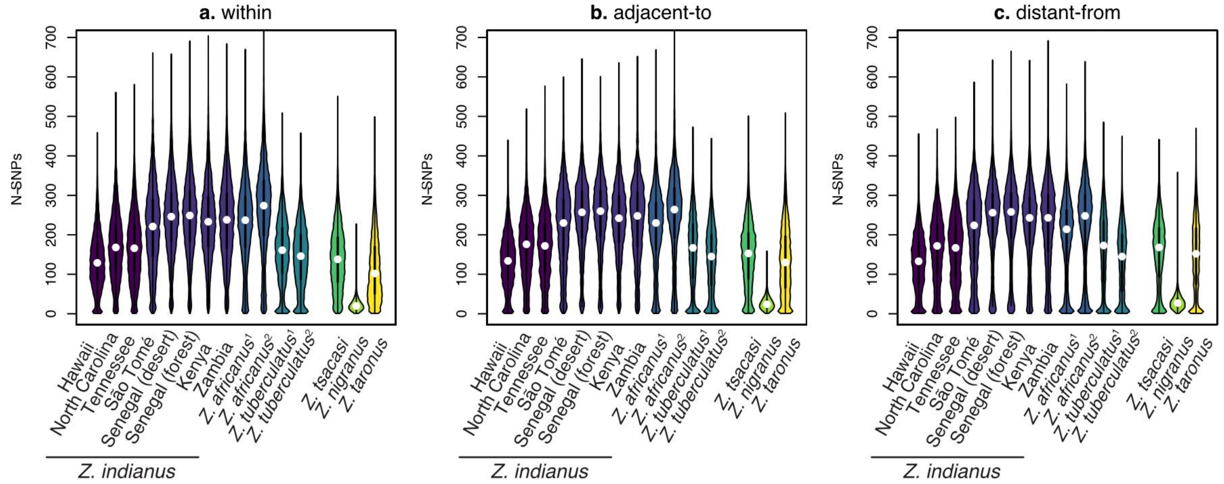

Figure S3. Genetic diversity (*S* == N-SNPs) summarized for windows either overlapping an annotated gene (**a**), within 5kb of an annotated gene (**b**), or greater than 5kb from the nearest annotated gene (**c**). *Z. africanus*[1] and *Z. tuberculatus*[1] were sampled from São Tomé, *Z. africanus*[2] from Kenya, and *Z. tuberculatus*[2] from Senegal (forest site).

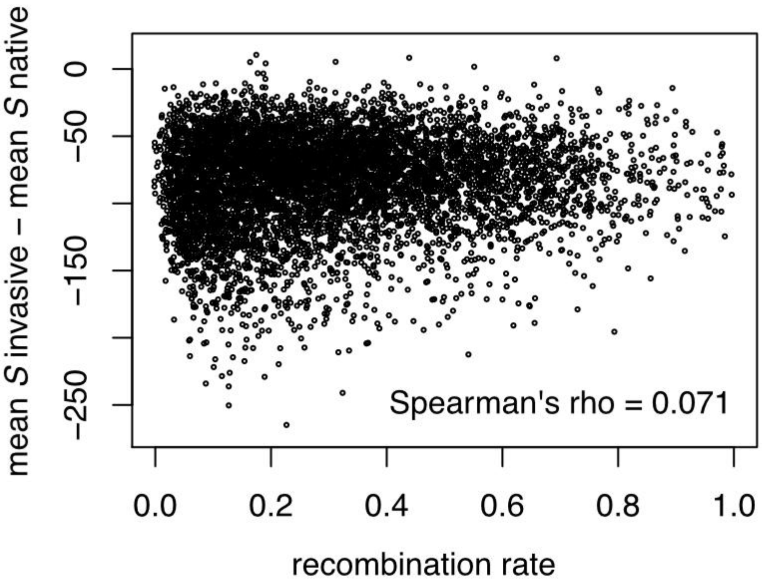

Figure S4. Correlation between the difference in amounts of genetic diversity (the number of segregating sites: *S*; mean across invasive populations - mean across native populations) and (population) recombination rate for *Z. indianus*.

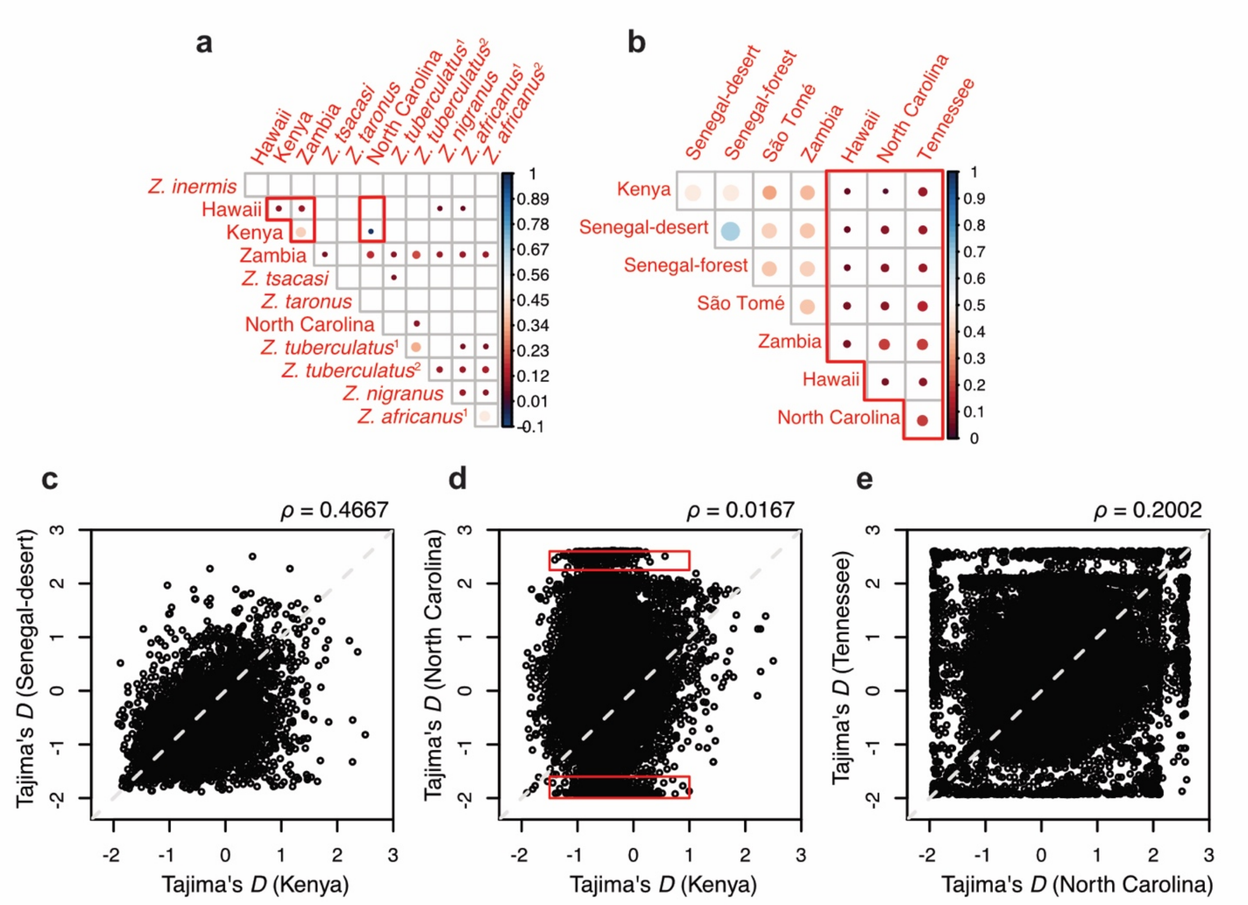

Figure S5. Tajima's *D* is weakly correlated or uncorrelated between different species of *Zaprionus* (**a**) and populations of *Z. indianus* (**b**). The strongest correlations in S (outside of populations of *Z. indianus* sampled from two locations in Senegal) were between geographically distant populations of *Z. indianus* in its native Africa (**c**) and the weakest correlation between *Z. indianus* populations was between a native and an invasive population (**d**). The two geographically proximate invasive populations of *Z. indianus* in North America showed a moderate correlation in Tajima's **D** across windows. Red polygons in **a** highlight comparisons between populations of *Z. indianus* and in **b** highlight comparisons between invasive populations. Red rectangles in panel **d** highlight genomic windows that show a pronounced shift in Tajima's *D* between the invasive population in North Carolina and the native population in Kenya.

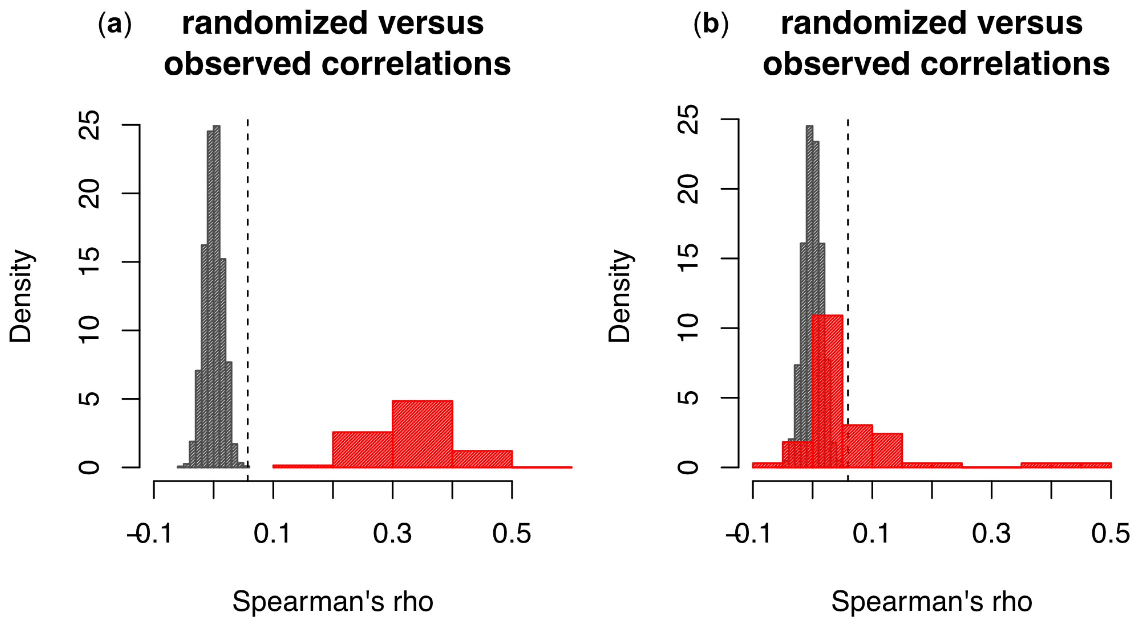

Figure S6. Randomized and observed correlation coefficients for genetic diversity (S; panel (a)) and Tajima's *D* (b). Grey histograms in each panel show the distribution of correlation coefficients generated when randomly selecting genomic windows that span BUSCO annotated genes in two species' genomes. Red histograms show observed correlation coefficients across all pairwise interspecific comparisons. The dashed vertical line in each panel represents the 95% tail of the randomized distribution.
